# Maternal employment patterns after childbirth and child mental health with 4–6 years of age

**DOI:** 10.3389/fpsyg.2025.1580373

**Published:** 2025-08-20

**Authors:** Deborah Wernecke, Raphael S. Peter, Stefanie Braig, Jon Genuneit, Dietrich Rothenbacher

**Affiliations:** ^1^Institute of Epidemiology and Medical Biometry, Ulm University, Ulm, Germany; ^2^German Center for Child and Adolescent Health (DZKJ), Partner Site Ulm, Ulm, Germany; ^3^Pediatric Epidemiology, Department of Pediatrics, Medical Faculty, Leipzig University, Leipzig, Germany; ^4^German Center for Child and Adolescent Health (DZKJ), Partner Site Leipzig, Leipzig, Germany

**Keywords:** maternal employment, mental health problems, quality of life, SDQ, KINDL

## Abstract

**Objectives:**

To assess potential associations between maternal employment after childbirth with subsequent child mental health problems.

**Methods:**

We analyzed 536 families of a prospective birth cohort to estimate trajectory classes of maternal employment 0–36 months postpartum. Multivariable linear regression models were employed to examine associations between (1) trajectory classes and (2) maternal employment status at 12 months postpartum with child mental health problems at 4, 5, and 6 years of age.

**Results:**

We identified five different trajectory classes of maternal employment after childbirth. For boys, trajectory classes 2 and 3 (characterized by relatively high maternal working hours/week) were associated with more mental health problems at ages 5 and 6 compared to class 1 (relatively low or no working hours/week). No associations were found for classes 4 (part-time after 2 years) and 5 (increasing and subsequently decreasing working hours/week) among boys. For girls, class 5 was associated with less mental health problems at ages 5, and 6 compared to class 1. Analysis of maternal employment status at 12 months postpartum showed less mental health problems for part-time employment at 12 months postpartum compared to no-employment among boys.

**Conclusion:**

Mixed results were found for maternal employment after childbirth and boys’ mental health depending on the measure used. No detrimental associations were found for girls.

## Introduction

The number of women with a young child and in paid employment is increasing in Western societies (Kopp et al., 2023; OECD Family Database, 2023; OECD, 2011; Han et al., 2008). This is a positive development that strengthens women’s emancipation and helps to maximize financial resources and hence family well-being (Greenstein, 1993; Huerta et al., 2011). However, these women frequently encounter social stigma, especially in Germany (Heffernan and Stone, 2021; Schreyögg, 2011; Amos, 2019; Dien, 2021; Diabaté et al., 2015), partly due to research findings suggesting that participating in the labor market while having a young child may be associated with child development if financial resources (and therefore goods and services) do not compensate for the minimized time, attention and emotional support available to the child (Kopp et al., 2023; Foster, 2002) (details see household economics framework) (Greenstein, 1993; Foster, 2002). There may also be a lack of support for working mothers from both the private and public sectors, leading to difficulties in dual roles and responsibilities (Poduval and Poduval, 2009; Carr et al., 1998; Bünning and Hipp, 2022; Boeckmann et al., 2015; Aarntzen et al., 2023). Unfortunately, many mothers do not have a choice. They have to contribute to the household income, or sometimes earn it alone, remain financially independent, and maintain career opportunities at the same level.

Data to explore the association between maternal employment patterns in early childhood and subsequent child heath are scarce as maternal employment is often assessed at a single point in time, and measures and social context, such as national policies for family leave, strongly vary across studies (Kopp et al., 2023; Huerta et al., 2011; Brooks-Gunn et al., 2010; Lucas-Thompson et al., 2010; Waldfogel, 2002; Hope et al., 2014; Berger et al., 2008; Salimiha et al., 2018). In addition, it is likely that possible negative direct effects of maternal employment on children’s mental health could be offset by possible positive indirect effects (Brooks-Gunn et al., 2010; Marks, 1977), for example through increased use of center-based care, increased maternal sensitivity, or increased maternal self-esteem and energy leading to better maternal mental health (Brooks-Gunn et al., 2010). These few points already indicate that the existing evidence is likely to show different results depending on the variables considered, the underlying theoretical framework, and the social context (Kopp et al., 2023; Friedline et al., 2021; Mooi-Reci and Wooden, 2022; Barnett, 2008; Masarik and Conger, 2017). Hence, an (evidence-based) “universal” advice for families with young children is difficult to give.

We analyzed in the context of a prospective birth cohort whether the maternal longitudinal employment pattern 36 months after childbirth was associated with child mental health problems at 4, 5, or 6 years of age. For comparison with the current literature, we also looked at an exposure assessed at a single time point: maternal employment status at 12 months postpartum. The analysis of data on paternal employment patterns has not been possible due to the incomparable quality of the data in the study.

## Methods

### Study design, study population, and ethical approval

The Ulm SPATZ Health Study (SPATZ) is a population-based longitudinal (birth) cohort study conducted in Ulm, southern Germany, which recruited newborns and their mothers during hospitalization after delivery at the Department of Gynecology and Obstetrics, University Hospital Ulm (at that time the only maternity hospital in Ulm). Baseline took place from April 2012 to May 2013 (overall response rate was 49%). Details are described elsewhere (Logan et al., 2016). Ethical approval was obtained from the Ethics Committee of the University of Ulm (no. 311/11).

### Sampling procedure and sample description

This study comprises a subsample of the SPATZ birth cohort: *n* = 557 (55.4%) children for whom at least one questionnaire was completed at age 4, 5, or 6 years for information on the outcomes were included in the analysis. For all these children, detailed longitudinal information on maternal employment was available up to 3 years after birth.

### Maternal employment as exposure variables

Hours per week of maternal employment were assessed in the 6 month questionnaire for the first 6 months postpartum, separately, and in 1, 2, and 3 year questionnaire for each of the 12 preceding months, separately. Mothers were clustered using a latent class model considering their working hours over the first 36 months postpartum. The model resulted in 5 *trajectory classes*, which were taken as the first exposure variable of interest (see statistical analysis and results below). As a second exposure variable of interest, *maternal employment status 1 year postpartum* was assessed at the 1-year follow-up with a separate question with four categories: full-time employment (≥35 h/week), part-time employment (<35 h/week), marginal part-time employment (mini-jobs or unregularly jobs with very few permitted monthly working hours; in Germany distinguished from part-time employment), and not-employed.

In this study, we exclusively utilized maternal data due to its superior quality compared to paternal data: The maternal data were collected from a comprehensive set of questionnaires from different follow up waves. In contrast, paternal data faced substantial selection bias attributed to non-participation (baseline data was available for 59% of children) and higher dropout rates. Furthermore, the variables related to employment patterns for fathers (or second mothers) were substantially limited, hindering comprehensive analysis (data on paternal working status was only available at the child’s age of 6 weeks for 54% of children).

### Outcome variables

Child mental health problems and child health-related quality of life with 4, 5, and 6 years of life were the main outcome variables of interest. Outcomes were assessed with two different validated questionnaires at each time point by parental reports: The Strengths and Difficulties Questionnaire (SDQ) measures a child’s mental health problems using an emotional and behavioral difficulties score. We used the validated German version of the SDQ (Goodman, 1997) which consists of 25 items covering five subscales (emotion, behavior, hyperactivity, peer relationships, and prosocial behavior). For the total emotional and behavioral difficulties score, all subscales except prosocial behavior are summed up (20 items); a higher score indicates more social and emotional difficulties. The KINDL-R questionnaire aims to measure a child’s health-related quality of life. We used the German version of the KINDL-R questionnaire (Ravens-Sieberer and Bullinger, 1998; the BELLA Study Group et al., 2008) which consists of 24 items covering six dimensions: family, physical well-being, emotional well-being, self-esteem, friends, and school. For the total score (outcome “health-related quality of life”), all 24 items are summed up and transformed to a 0–100 scale; higher values represent better health-related quality of life.

### Confounding variables and effect measure modification

For the identification of confounding variables, we developed a Directed Acyclic Graph (DAG) based on literature (Kopp et al., 2023; Han et al., 2008; Brooks-Gunn et al., 2010; Waldfogel, 2002; Berger et al., 2008; Hondralis and Kleinert, 2021; Dex et al., 1998; Gustafsson et al., 1996; Hanel and Riphahn, 2012) (Supplementary Figure S1). We used DAGitty (Textor et al., 2017) to find a set of variables that is minimal and sufficient to deconfound the association between the exposure and the outcome in our stated DAG (the minimal sufficient adjustment set) (Williamson et al., 2014). The minimal sufficient adjustment set was used for adjustment in the multivariable linear regression models.

Evaluation of potential effect measure modification between maternal employment and child gender was done, since the association between parental employment and child mental health problems might be moderated by child gender (McMunn et al., 2012). In case of a significant interaction, models will be run separately for boys and girls, to align with the findings of age- and sex-specific norm values for the German total SDQ-score (Janitza et al., 2020). We did not test effect modification for maternal occupation or child temperament, as they are rather unlikely to moderate the association (Brooks-Gunn et al., 2010; Hondralis and Kleinert, 2021).

#### Maternal and paternal mental health and chronic stress

Maternal mental health at birth was indexed by the German version of the Hospital Anxiety and Depression Scale (HADS). The HADS questionnaire is a validated 14-item screening measure with two subscales assessing symptoms of anxiety and depression in the last week prior to assessment (Herrmann, 1997; Petermann, 2011). The questionnaire is also validated in the German language (Petermann, 2011; Hermann-Lingen et al., 2011).

Chronic maternal and paternal stress was measured using the Screening Scale of the Trier Inventory of Chronic Stress (SSCS-TICS) (Schulz and Schlotz, 1999). It aims to assess chronic concerns, work overload, excessive demands, lack of social recognition, and social stress 3 months prior to assessment. Maternal data was assessed at birth, and paternal data 6 weeks postpartum.

#### Type of childcare by time points after childbirth

The type of childcare other than maternal care was assessed at 6 months, 1, 2, and 3 years. Based on the literature (Berger et al., 2008), four categories of care were distinguished: paternal care, center-based care, relative care, and non-relative care (details see Supplementary Methods).

### Statistical analysis

We used general latent class mixed models (R-package “lcmm”) for the identification of classes of maternal employment trajectories between 0 to 36 months after childbirth (details see Supplementary Methods). We conducted descriptive statistics for baseline characteristics in the whole SPATZ cohort and for the analyzed study sample.

Multivariable linear regression analysis was applied to estimate the potential association between (1) *trajectory classes* of maternal employment after childbirth, and (2) *maternal employment status 1 year postpartum* with child mental health with 4, 5, and 6 years of age, respectively. *p*-values and 95% confidence intervals (CI) were calculated based on the robust standard errors. A complete case analysis was conducted, as only a relatively small percentage of the data on exposure was missing (15.8%) and the analysis cohort can be well characterized by Table 1, which makes the potential for selection bias assessable. The analysis was performed using SAS® 9.4 (The SAS Institute, Cary, NC, USA). The general latent class mixed models were calculated by using R (R Foundation for Statistical Computing, Vienna, Austria).

**Table 1:** Baseline characteristics of all mothers (*n* = 970) and those included in the statistical analysis (*n* = 536).

	*n*	All mothers *n* = 970	*n*	Mothers included in the statistical analysis *n* = 536^a^
Age at birth, mean (SD), (min, max)	969	32.7 (4.8), (18.4, 54.3)	536	33.4 (4.5), (20.8, 54.3)
Nationality, *N* (%)				
German	962	857 (89.1)	535	496 (92.7)
Non-German		105 (10.9)		39 (7.3)
Maternal educational attainment, *N* (%)				
≥12 years	951	561 (59.0)	530	372 (70.2)
10–11 years		297 (31.2)		134 (25.3)
≤9 years		93 (9.8)		24 (4.5)
Paternal educational attainment, *N* (%)				
≥12 years	575	395 (68.7)	387	278 (71.8)
<12 years		180 (31.3)		109 (28.2)
Maternal employment status before maternity leave, *N* (%)				
Full-time (≥35 h/week)	970	505 (52.1)	536	308 (57.5)
Part-time (<35 h/week)		221 (22.8)		120 (22.4)
Apprenticeship		8 (0.8)		3 (0.6)
Marginal part-time employment		45 (4.6)		20 (3.7)
Not employed		178 (18.4)		83 (15.5)
Missing		13 (1.3)		2 (0.4)
Maternal Hospital Anxiety and Depression Scale (HADS)^b^, mean (SD), median (Q1, Q3)	957	7.3 (5.0), 6.0 (4.0, 10.0)	534	6.8 (4.4), 6.0 (3.0, 9.0)
HADS_A (Anxiety) ≥ 8, *N* (%)	958	176 (18.4)	534	82 (15.4)
HADS_D (Depression) ≥ 8, *N* (%)	960	46 (4.5)	534	17 (3.2)
Maternal chronic stress (TICS_t), mean (SD), median (Q1, Q3)	959	49.2 (10.1), 50.0 (43.0, 56.0)	534	48.6 (9.8), 49.5 (42.0, 56.0)
TICS_t > 60 (noticeable), *N* (%)	959	131 (13.4)	534	63 (11.8)
Paternal TICS_t, mean (SD), median (Q1, Q3)	573	50.1 (10.0), 50.0 (44.0, 57.0)	385	49.6 (9.9), 50.0 (43.0, 56.0)
TICS_t > 60 (noticeable), *N* (%)	573	97 (16.9)	385	57 (14.8)
Mothers who had a second child before birth of study child	970	228 (23.5)	536	182 (34.0)
Mothers who got a second child 0–36 months after birth of study child (only for mothers participating 0–36 months)	625	99 (15.8)	502	88 (17.5)

Ulm SPATZ Health Study, Germany. ^a^ only mothers still participating in the 4-year-follow-up and questionnaire data of the outcome variable (mental health of child assessed at the 4 year-follow-up) were available. ^b^ summary scores on each subscale range from 0 to 21. A score below 8 is considered normal, a score between 8 and 10 indicates moderate levels of symptoms, and a score between 11 and 21 indicates severe levels of symptoms.

## Results

### Descriptive results

The baseline characteristics of the SPATZ cohort (*n* = 970 mothers) and the analyzed sample (n = 536 mothers) are described in Table 1. The analyzed sample comprised more mothers with higher educational attainment (70.2% vs. 59.0%), German nationality (92.7% vs. 89.1%), and full-time workers before the birth of the study child (57.5% vs. 52.1%) compared to all participants. The mean maternal age at childbirth was 33.4 years (SD 4.5) in the analyzed sample.

Table 2 shows descriptive data for mothers’ employment status 1, 2 and 3 years after childbirth. The proportion of mothers in full- and part-time employment increased from year 1 to year 2, consequently the proportion of mothers not employed decreased from 64.1 to 32.4%. Between years 2 and 3 these percentages remained more or less the same. Descriptive data for maternal weekly working hours 0–36 months after childbirth is shown in Supplementary Table S1. This data was used to identify *trajectory classes* of maternal employment after childbirth. Supplementary Table S2 shows descriptive data for the type of childcare other than by the mother in the first 3 years of life. Supplementary Tables S3, S4 show the descriptive results of outcome variables in children with 4, 5, and 6 years of age, overall and by respective exposure variables.

**Table 2:** Maternal employment status 6 months, 1, 2, and 3 years after childbirth.

	*n*	6 months *n* = 768 mothers	*n*	1 year *n* = 726 mothers	*n*	2 years *n* = 694 mothers	*n*	3 years *n* = 625 mothers
		*N* (%)		*N* (%)		*N* (%)		*N* (%)
Having any kind of employment since childbirth	766	101 (13.2)	710	250 (35.2)	678	453 (66.8)	597	398 (66.7)
Full-time (≥35 h/week)	no data		726	58 (8.0)	694	80 (11.5)	625	80 (12.8)
Part-time (<35 h/week)				119 (16.4)		293 (42.2)		264 (42.2)
Apprenticeship				4 (0.6)		3 (0.4)		3 (0.5)
Marginal part-time employment				69 (9.5)		79 (11.4)		52 (8.3)
Not employed				465 (64.1)		225 (32.4)		199 (31.8)
Missing				11 (1.5)		12 (1.7)		28 (4.5)

Ulm SPATZ Health Study, Germany.

#### Confounding variables

The minimal sufficient adjustment set derived from the DAG comprised the following 12 variables: maternal nationality (German vs. non-German), maternal highest educational attainment (<12 yrs. school education vs. ≥ 12 yrs.), paternal highest educational attainment (<12 yrs. vs. ≥ 12 yrs.), mother had a second child before the birth of the study child, mother got a second child within 36 months after the birth of the study child, maternal HADS-score at baseline, maternal TICS-score at baseline, paternal TICS-score 6 weeks after childbirth, childcare done by the partner at child age 12 months (≥10 h/week vs. < 10 h/week), childcare done by center-based care at child age 12 months (≥10 h/week vs. < 10 h/week), childcare done by relatives at child age 12 months (≥10 h/week vs. < 10 h/week), childcare done by non-relatives at child age 12 months (≥10 h/week vs. < 10 h/week). In order to enhance the transparency of the results, the variables were introduced into the model in two distinct steps (adjustment “a” and “b”).

Evidence of effect modification between maternal employment and child gender was found in the final models, therefore the analysis was done for boys and girls separately.

### Analytical results

#### Trajectories of maternal employment

**Figure 1:**
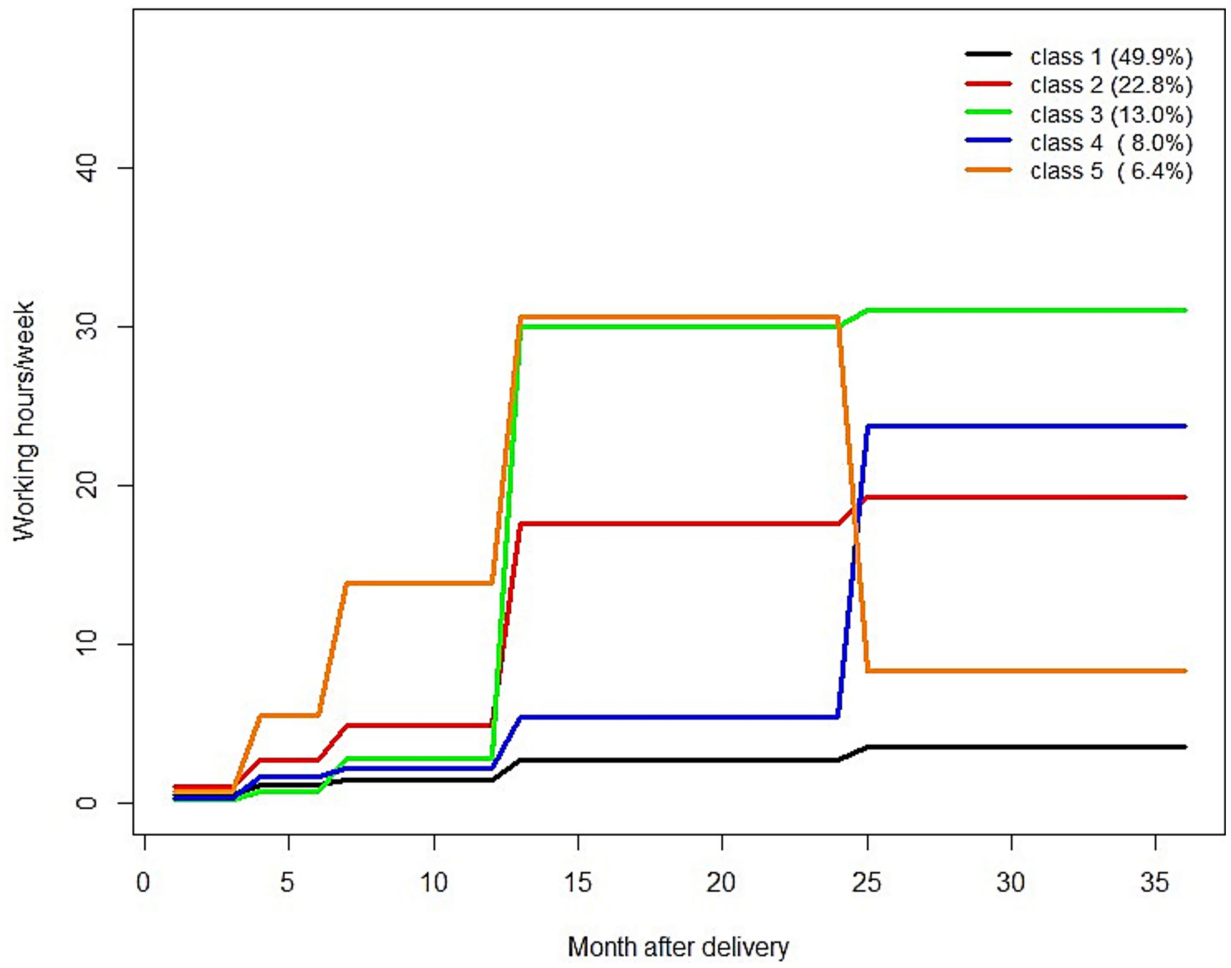
Trajectory classes of maternal employment 0–36 months after childbirth; ordered by group size/number of mothers in trajectory classes. Class 1: “no or very low % of employment,” class 2: “part-time after 1 year,” class 3: “full time after 1 year,” class 4: “part-time after 2 years,” class 5: “rapidly increasing – decreasing”.

We identified five *trajectory classes* (weekly working hours per month) for maternal employment 0–36 months after childbirth in *n* = 817 mothers (Figure 1 and Supplementary Figure S3). Class 1 is characterized by “no or very low % of employment” and comprised *n* = 408 mothers, class 2, characterized by “part-time after 1 year” *n* = 186 mothers, class 3, characterized by “full time after 1 year” *n* = 106 mothers, class 4, characterized by “part-time after 2 years” *n* = 65 mothers, and class 5, characterized by “rapidly increasing – decreasing” *n* = 52 mothers. The analysis sample, comprising mother–child pairs with complete data on exposure and outcome, was distributed across the trajectory classes as follows: class 1: 44.7% (*n* = 249), class 2: 25.1% (*n* = 140), class 3: 14.4% (*n* = 80), class 4: 9.0% (*n* = 50), class 5: 6.8% (*n* = 38).

Table 3 shows the results of the adjusted linear regression models (adjustments a, and b) estimating the association between maternal employment (trajectory classes, and employment status 12 months after childbirth) and child total SDQ-scores with 4, 5, and 6 years of age by gender. Table 4 shows the results for the same statistical analysis but with the outcome KINDL-score, instead of the SDQ-score.

**Table 3:** Associations between maternal employment after childbirth (*n* = 5 trajectory classes, and working status 1 year after childbirth) and mental health problems in children aged 4, 5, and 6 indexed with the total SDQ-score by gender (results of adjusted linear regression models).

	Difference in means (95% CI) in total SDQ-scores by child age								
	*n*	4 years	*p*	*n*	5 years	*p*	*n*	6 years	*p*
Boys									
Trajectory classes ^a^	192			170			152		
Class 1	77	ref.		67	ref.		59	ref.	
Class 2	49	−0.78 (−2.34, 0.78)	0.33	47	0.54 (−1.22, 2.30)	0.55	41	0.46 (−1.49, 2.41)	0.64
Class 3	28	0.48 (−1.44, 2.41)	0.62	26	1.06 (−0.99, 3.12)	0.31	24	**2.63 (0.26, 4.99)**	**0.03**
Class 4	20	−1.11 (−3.57, 1.35)	0.38	12	−0.80 (−2.94, 1.34)	0.46	14	−0.60 (−2.92, 1.71)	0.61
Class 5	18	−1.55 (−3.71, 0.61)	0.16	18	0.55 (−1.95, 3.06)	0.67	14	0.39 (−1.73, 2.50)	0.72
Trajectory classes ^b^	190			168			151		
Class 1	77	ref.		67	ref.		59	ref.	
Class 2	48	0.24 (−1.26, 1.73)	0.75	46	**1.81 (0.33, 3.30)**	**0.02**	41	1.27 (−0.46, 2.99)	0.15
Class 3	27	1.26 (−0.87, 3.40)	0.25	25	**2.14 (0.37, 3.90)**	**0.02**	23	**2.77 (0.67, 4.87)**	**0.01**
Class 4	20	−0.01 (−2.38, 2.34)	0.99	12	0.33 (−1.92, 2.59)	0.77	14	0.05 (−2.02, 2.11)	0.96
Class 5	18	−0.34 (−2.41, 1.73)	0.74	18	1.92 (−0.38, 4.21)	0.10	14	1.67 (−0.41, 3.75)	0.12
Girls									
Trajectory classes ^a^	183			166			154		
Class 1	83	ref.		72	ref.		67	ref.	
Class 2	42	0.37 (−1.25, 1.99)	0.65	39	0.39 (−1.53, 2.30)	0.69	34	−0.71 (−2.54, 1.12)	0.45
Class 3	27	−0.23 (−2.33, 1.87)	0.83	25	0.26 (−1.77, 2.28)	0.81	27	−1.58 (−3.61, 0.45)	0.13
Class 4	19	0.61 (−2.37, 3.58)	0.69	18	−0.73 (−2.64, 1.19)	0.46	15	−1.33 (−3.56, 0.91)	0.24
Class 5	12	−0.33 (−2.37, 1.70)	0.75	12	−1.79 (−3.63, 0.04)	0.06	11	**−3.39 (−5.34, −1.45)**	**<0.001**
Trajectory classes ^b^	179			163			151		
Class 1	83	ref.		72	ref.		**67**	ref.	
Class 2	40	0.11 (−1.59, 1.81)	0.90	38	0.72 (−1.42, 2.86)	0.51	**32**	−0.70 (−2.59, 1.19)	0.47
Class 3	27	−0.003 (−2.23, 2.22)	0.99	25	0.53 (−1.63, 2.69)	0.63	**27**	−1.47 (−3.40, 0.45)	0.13
Class 4	17	0.82 (−2.27, 3.92)	0.60	16	−1.06 (−3.09, 0.97)	0.31	14	−1.33 (−3.51, 0.84)	0.23
Class 5	12	−0.44 (−2.67, 1.78)	0.70	12	**−2.17 (−4.26, −0.07)**	**0.04**	11	**−3.84 (−6.14, −1.53)**	**0.001**
Boys									
Employment at 12 m. ^a^	185			165			147		
Not employed	140	ref.		125	ref.		111	ref.	
Marginal part-time	11	−1.10 (−3.15, 0.95)	0.29	9	−0.47 (−3.85, 2.92)	0.79	9	−0.03 (−2.77, 2.70)	0.98
Part-time	21	**−1.88 (−3.65, −0.12)**	**0.04**	20	−1.26 (−3.34, 0.82)	0.24	16	−0.06 (−2.74, 2.63)	0.97
Full-time	13	0.35 (−2.23, 2.94)	0.79	11	1.10 (−1.75, 3.95)	0.45	11	1.63 (−0.22, 3.49)	0.08
Employment at 12 m. ^b^	183			163			146		
Not employed	139	ref.		123	ref.		110	ref.	
Marginal part-time	11	−0.78 (−3.15, 1.58)	0.52	9	−0.03 (−2.81, 2.75)	0.99	9	0.24 (−2.07, 2.55)	0.84
Part-time	21	**−2.10 (−3.52, −0.68)**	**0.004**	20	−0.96 (−2.77, 0.85)	0.30	16	0.49 (−2.07, 3.04)	0.71
Full-time	12	0.25 (−1.65, 2.14)	0.80	10	0.98 (−1.18, 3.14)	0.37	11	1.52 (−0.86, 3.91)	0.21
Girls									
Employment at 12 m. ^a^	177			160			146		
Not employed	109	ref.		100	ref.		92	ref.	
Marginal part-time	15	1.08 (−2.00, 4.16)	0.49	12	3.43 (−0.27, 7.13)	0.07	12	1.40 (−2.36, 5.16)	0.46
Part-time	38	0.23 (−1.35, 1.80)	0.78	32	−0.08 (−1.64, 1.49)	0.93	27	−0.38 (−2.25, 1.49)	0.69
Full-time	15	−0.25 (−2.60, 2.10)	0.83	16	0.32 (−1.69, 2.32)	0.76	15	−1.73 (−3.93, 0.46)	0.12
Employment at 12 m. ^b^	173			157			143		
Not employed	106	ref.		98	ref.		90	ref.	
Marginal part-time	15	0.78 (−2.55, 4.10)	0.65	12	3.37 (−0.52, 7.26)	0.09	12	1.57 (−2.12, 5.26)	0.41
Part-time	37	0.21 (−1.68, 2.10)	0.83	31	0.57 (−1.23, 2.36)	0.54	26	0.02 (−2.25, 2.28)	0.99
Full-time	15	−0.13 (−2.63, 2.37)	0.92	16	0.57 (−1.54, 2.69)	0.60	15	−1.82 (−4.09, 0.44)	0.11

Ulm SPATZ Health Study, Germany. ^a^ Adjusted for maternal nationality (German vs. non-German), maternal highest educational attainment (<12 yrs. school education vs. ≥ 12 yrs.), paternal highest educational attainment (<12 yrs. vs. ≥ 12 yrs.), mother had a second child before birth of study child, mother got second child within 36 months after the birth of study child. ^b^ adjusted as a, and additionally for maternal HADS-score at baseline, maternal TICS-score at baseline, paternal TICS-score at baseline, child-care done by partner with 12 months (≥10 h/week vs. < 10 h/week), child-care done by center-based care with 12 months (≥10 h/week vs. < 10 h/week), child-care done by relatives with 12 months (≥10 h/week vs. < 10 h/week), child-care done by non-relatives with 12 months (≥10 h/week vs. < 10 h/week). Results from the crude models are shown in Supplementary Table S5. Results of the adjustment variables from the fully adjusted models (i.e., adjustment b) are shown in Supplementary Tables S7, S8. SDQ-scores: lower scores indicate fewer social and emotional difficulties. Class 1: “no or very low % of employment,” class 2: “part-time after 1 year,” class 3: “full time after 1 year,” class 4: “part-time after 2 years,” class 5: “rapidly increasing – decreasing.” Please see also Figure 1. Full-time: ≥35 h/week (includes *n* = 2 mothers doing an apprenticeship); part-time: <35 h/week; marginal part-time: employment such as mini-jobs or irregular jobs with very few working hours; CI: Confidence intervall; m: months; yrs.: years. *p*-values < 0.05 are in bold and signify a statistically significant difference.

**Table 4:** Associations between maternal employment after childbirth (*n* = 5 trajectory classes, and working status 12 months after childbirth) and health-related quality of life in children aged 4, 5, and 6 indexed with the total KINDL-score by gender (results of adjusted linear regression models).

	Difference in means (95% CI) in total KINDL-scores by child age								
	*n*	4 years (T6)	*p*	*n*	5 years (T7)	*p*	*n*	6 years (T8)	*p*
Boys									
Trajectory classes ^a^	188			167			136		
Class 1	84	ref.		64	ref.		54	ref.	
Class 2	42	−0.46 (−3.19, 2.28)	0.74	46	2.23 (−0.79, 5.26)	0.15	35	−0.69 (−3.80, 2.42)	0.66
Class 3	27	1.29 (−1.92, 4.51)	0.43	27	2.66 (−1.13, 6.44)	0.17	19	−3.00 (−6.62, 0.62)	0.10
Class 4	18	0.29 (−3.09, 3.67)	0.87	12	0.92 (−2.97, 4.81)	0.64	14	0.49 (−3.51, 4.48)	0.81
Class 5	12	2.06 (−1.47, 5.58)	0.25	18	0.81 (−3.33, 4.95)	0.70	14	1.91 (−2.24, 6.05)	0.37
Trajectory classes ^b^	186			165			135		
Class 1	74	ref.		64	ref.		54	ref.	
Class 2	49	−2.04 (−4.42, 0.33)	0.09	45	0.39 (−2.29, 3.07)	0.78	35	**−3.14 (−5.99, −0.29)**	**0.03**
Class 3	27	−0.01 (−3.00, 2.98)	0.99	26	1.88 (−1.50, 5.26)	0.28	18	**−5.45 (−8.90, −2.00)**	**0.002**
Class 4	19	−2.40 (−5.74, 0.94)	0.16	12	−0.98 (−5.37, 3.40)	0.66	14	−2.44 (−5.90, 1.03)	0.17
Class 5	17	0.15 (−2.87, 3.17)	0.92	18	−1.40 (−4.95 2.15)	0.44	14	−0.77 (−4.75, 3.21)	0.70
Girls									
Trajectory classes ^a^	183			161			131		
Class 1	84	ref.		70	ref.		58	ref.	
Class 2	42	1.59 (−1.14, 4.33)	0.25	36	0.32 (−2.73, 3.37)	0.84	28	−0.23 (−3.53, 3.07)	0.89
Class 3	27	1.83 (−1.55, 5.20)	0.29	25	1.10 (−2.61, 4.81)	0.56	25	−0.27 (−3.68, 3.15)	0.88
Class 4	18	0.68 (−3.26, 4.62)	0.74	18	1.83 (−1.76, 5.42)	0.32	12	0.24 (−3.93, 4.42)	0.91
Class 5	12	2.46 (−1.21, 6.14)	0.19	12	2.30 (−1.12, 5.73)	0.19	8	2.18 (−2.92, 7.27)	0.40
Trajectory classes ^b^	179			158			128		
Class 1	84	ref.		70	ref.		58	ref.	
Class 2	40	2.23 (−0.60, 5.05)	0.12	35	0.11 (−2.74, 2.96)	0.94	26	0.61 (−2.55, 3.77)	0.71
Class 3	27	1.70 (−1.97, 5.37)	0.36	25	0.43 (−3.37, 4.23)	0.83	25	0.10 (−3.54, 3.73)	0.96
Class 4	16	1.37 (−2.40, 5.14)	0.48	16	2.91 (−0.24, 6.06)	0.07	11	1.18 (−2.62, 4.98)	0.54
Class 5	12	2.58 (−1.94, 7.10)	0.26	12	3.22 (−0.60, 7.03)	0.10	8	3.45 (−2.82, 9.71)	0.28
Boys									
Employment at 12 m. ^a^	182			162			132		
Not employed	135	ref.		122	ref.		101	ref.	
Marginal part-time	11	−1.90 (−5.54, 1.74)	0.31	8	−1.77 (−6.33, 2.78)	0.45	8	−1.26 (−4.43, 1.91)	0.43
Part-time	22	0.52 (−2.27, 3.32)	0.71	20	1.32 (−2.22, 4.87)	0.46	14	0.10 (−3.23, 3.42)	0.96
Full-time	14	−1.47 (−5.65, 2.72)	0.49	12	−0.54 (−3.85, 2.77)	0.75	9	1.06 (−4.65, 6.77)	0.72
Employment at 12 m. ^b^	180			160			131		
Not employed	11	ref.		121	ref.		100	ref.	
Marginal part-time	22	−2.61 (−5.85, 0.64)	0.12	8	−2.82 (−6.17, 0.52)	0.10	8	−0.21 (−3.33, 2.90)	0.89
Part-time	13	0.44 (−2.40, 3.27)	0.76	20	−0.13 (−4.16, 3.89)	0.95	14	−1.74 (−5.21, 1.73)	0.33
Full-time	11	−0.72 (−3.36, 1.93)	0.60	11	−0.85 (−4.54, 2.84)	0.65	9	0.01 (−4.79, 4.82)	0.99
Girls									
Employment at 12 m. ^a^	176			155			124		
Not employed	109	ref.		96	ref.		80	ref.	
Marginal part-time	14	−2.56 (−6.51, 1.39)	0.20	11	−3.80 (−9.42, 1.82)	0.18	11	−3.85 (−7.79, 0.09)	0.06
Part-time	37	0.28 (−2.44, 2.99)	0.84	32	1.19 (−1.97, 4.35)	0.46	22	1.30 (−2.36, 4.97)	0.49
Full-time	16	1.54 (−1.75, 4.82)	0.36	16	1.34 (−1.96, 4.65)	0.43	11	−2.80 (−7.42, 1.81)	0.23
Employment at 12 m. ^b^	172			152			121		
Not employed	106	ref.		94	ref.		78	ref.	
Marginal part-time	14	−2.27 (−6.26, 1.72)	0.27	11	−3.34 (−8.91, 2.24)	0.24	11	−3.43 (−7.48, 0.62)	0.097
Part-time	36	0.15 (−3.28, 3.58)	0.94	31	0.62 (−2.88, 4.12)	0.73	21	1.59 (−2.87, 6.05)	0.48
Full-time	16	1.13 (−2.48, 4.75)	0.54	16	0.71 (−2.60, 4.03)	0.67	11	−2.11 (−6.98, 2.76)	0.40

Ulm SPATZ Health Study. ^a^ adjusted for maternal nationality (German vs. non-German), maternal highest educational attainment (<12 yrs. school education vs. ≥ 12 yrs.), paternal highest educational attainment (<12 yrs. vs. ≥ 12 yrs.), mother had a second child before birth of study child, mother got second child within 36 months after the birth of study child. ^b^ adjusted as a, and additionally for maternal HADS-score at baseline, maternal TICS-score at baseline, paternal TICS-score at baseline, child-care done by partner with 12 months (≥10 h/week vs. < 10 h/week), child-care done by center-based care with 12 months (≥10 h/week vs. < 10 h/week), child-care done by relatives with 12 months (≥10 h/week vs. < 10 h/week), child-care done by non-relatives with 12 months (≥10 h/week vs. < 10 h/week). Results from the crude models are shown in Supplementary Table S6. Results of adjustment variables from fully adjusted models (i.e. adjustment b) are shown in Supplementary Tables S9, S10. KINDL-scores: higher scores indicate better health-related quality of life. Class 1: “no or very low % of employment,” class 2: “part-time after 1 year,” class 3: “full time after 1 year,” class 4: “part-time after 2 years,” class 5: “rapidly increasing – decreasing.” Please see also Figure 1. Full-time: ≥35 h/week (includes *n* = 2 mothers doing an apprenticeship); part-time: <35 h/week; marginal part-time: employment such as mini-jobs or irregular jobs with very few working hours; CI: Confidence intervall; m: months; yrs.: years. *p*-values < 0.05 are in bold and signify a statistically significant difference.

### Results in boys

Classes 2 (“part-time after 1 year”), and 3 (“full time after 1 year”) were both compared to class 1 (“no or very low % of employment”) associated with more social and emotional difficulties in boys at age 5 [difference in means: 1.81 (95% CI: 0.33, 3.30), 2.14 (0.37, 3.90)]. Additionally, class 3 (“full time after 1 year”) was found to be associated with the same outcome at 6 years [2.77 (0.67, 4.87)]. Point estimates for the associations between trajectory classes 2, and 3 with child outcomes at 4 years of age were pointing in the same direction, but failed to be statistically significant, possibly due to the limited sample size. The same was found for the associations between class 2 and child outcomes at child age 6 years. There was also a negative association with health-related quality of life at 6 years for classes 2 and 3 vs. 1 [−3.14 (−5.99, −0.29) and −5.45 (−8.90, −2.00), respectively]. Part-time employment at 12 months postpartum was associated with fewer social and emotional difficulties at 4 years compared to no employment at 12 months postpartum [−2.10 (−3.52, −0.68)].

### Results in girls

Class 5 (“rapidly increasing – decreasing”) compared to class 1 (“no or very low % of employment”) was associated with fewer social and emotional difficulties at ages 5 and 6 (−2.17 (95% CI −4.26, −0.07) and −3.84 (−6.14, −1.53), respectively). Both, class 2 (“part-time after 1 year”) and 3 (“full time after 1 year”) compared to class 1 (“no or very low % of employment”) were not associated with social and emotional difficulties or with health-related quality of life among girls. Also, neither part-time nor full-time employment at 12 months postpartum, compared with no employment, was associated with the above outcomes.

## Discussion

In this prospective birth cohort study including 536 families, we estimated five different classes of maternal employment trajectories 0–36 months after childbirth and their possible associations with social and emotional outcomes in children aged 4–6 years. Our findings indicate no detrimental association between maternal employment in the first 3 years postpartum and girl’s mental health. Mixed results were found for boys, depending on the exposure assessed. Trajectory classes 2 (“part-time after 1 year”), and 3 (“full time after 1 year”) compared to class 1 (“no or very low % of employment”) were associated with more mental health problems at ages 5 and 6, but the sole assessment of maternal employment status 1 year postpartum showed fewer mental health problems in boys with part-time employed mothers at 12 months postpartum compared to boys with non-employed mothers at 12 months postpartum. These mixed findings highlight the complexity of the issue, particularly with regard to exposure assessment, and suggest the possibility of effect modification by gender.

Mothers in trajectory classes 2 (“part-time after 1 year”), and 3 (“full time after 1 year”) had worked relatively few hours in the workforce during the first 12 months postpartum, whereas mothers in class 5 had worked relatively many hours in the first 12 months. Other studies have suggested that an early return to work by mothers, in particular in the first 12 months postpartum may be detrimental to the child’s mental health (Kopp et al., 2023; Lucas-Thompson et al., 2010). Our results for trajectory classes do not support the specific conclusion regarding a “sensible” exposure window in the first 12 months postpartum. This is because among girls class 5 (including mothers working in the first 12 months) showed decreased mental health problems, whereas among boys, classes 2, and 3 (both starting work after 12 months) showed increased mental health problems. Though, our results among boys seem to support the possibility that among boys it is early, rather than later, maternal employment that may be associated with boys’ mental health (Brooks-Gunn et al., 2010; Lucas-Thompson et al., 2010; Berger et al., 2008; Francesconi and Ermisch, 2000; Hadzic et al., 2013). The reason is that class 4 (“part-time after 2 years”) was not associated with mental health problems among boys, however, classes 2 (“part-time after 1 year”), and 3 (“full time after 1 year”) were. The associations found among girls are consistent with findings from a large nationally representative cohort of British children born in the new millennium (McMunn et al., 2012). The authors found no detrimental effect of maternal employment on child mental health, and even suggested a positive effect on the mental health of 5-year-old girls (McMunn et al., 2012). Meta-analyses by Lucas-Thompson et al. (2010) and Kopp et al. (2023) revealed that maternal employment during the second and third years after childbirth may be associated with higher subsequent achievement and better mental health among children. Further research supports these findings (Kopp et al., 2023; Lucas-Thompson et al., 2010; Hope et al., 2014; Salimiha et al., 2018; Nomaguchi, 2006). The associations are in particular found if psychological and socioeconomic benefits of the employment outweigh the reduced mother–child interactions and the long hours in non-parental care (Kopp et al., 2023; Greenstein, 1993; Hope et al., 2014; Nomaguchi, 2006).

### Employment at 12 months

Our results suggest that maternal part-time vs. no employment at 1 year postpartum was associated with fewer social and emotional difficulties at 4 years only in boys. Similarly, Brooks-Gun et al. found that early part-time employment may have a positive indirect effect on externalizing problems, through differences in the home environment and maternal sensitivity (Brooks-Gunn et al., 2010). In a meta-analysis, Kopp et al. (2023) suggested that part-time employment, longer employment, and returning to work later than 12 months postpartum may be particularly beneficial for a child’s mental health (Kopp et al., 2023). Other studies support this conclusion (Nomaguchi, 2006; Fiori, 2020). However, as noted above, we also found that for boys, trajectory classes 2 (“part-time after 1 year”), and 3 (“full time after 1 year”) were associated with adverse outcomes at ages 5 and 6. These results appear somewhat contradictory, but show that the assessment of employment status at a single point in time (at 12 months postpartum) was associated with a positive effect at age 4 (near-term outcome), but no longer at ages 5, and 6 years, whereas assessment of employment trajectories (reflecting maternal employment patterns 0–36 months postpartum) showed effects in the opposite direction for more distal outcomes. Thus, our analysis indicates that appropriate definitions of the exposure and the exposure-outcome window are of crucial importance. I.e. the assessment of a “one-point-in-time” exposure may be reliable for an outcome temporally close to it. However, since maternal working patterns may change over time, a longer exposure window (e.g., employment trajectories) is necessary for a valid analysis of more distal outcomes. This finding is in alignment with the observations made in a meta-analysis by Kopp et al. (2023), who emphasized that the associations between maternal employment and child mental health are contingent on the operationalization of maternal employment, the investigated outcome, and the characteristics of the study and sample.

We believe that using trajectory classes as an exposure variable to capture maternal employment over a three-year period is superior to using an exposure variable that measures maternal employment status at only one point in time. In our analysis, we calculated trajectories based on 36 data points per individual as we had employment data for each month. The difference in results found by exposure variable suggests that future studies need to identify and capture a reliable exposure.

### Moderators, mediators, and adjustment variables

Our finding that child gender moderates the association between maternal employment and child mental health problems is somewhat consistent with the current literature (Brooks-Gunn et al., 2010; McMunn et al., 2012). Brooks-Gun et al. reported that in the few cases where effect modification occurred, the association was more detrimental for boys than for girls (analyzing *n* = 900 children) (Brooks-Gunn et al., 2010). However, Hope et al., including *n* = 11,538 children, found no effect modification between child gender and maternal employment (Hope et al., 2014). The difference might result from the relatively low sample size, the adjustment variables included in the statistical model, or the age of the children when the outcome was assessed (7 years vs. 4–6 years). In our study (as well as in the studies cited), reporting bias (perception bias) is possible: Interestingly, maternal employment status affects parents’ perceptions of their child’s mental health differently for boys and girls, and may therefore contribute to gender differences in the effects of maternal employment on child mental health (Bronfenbrenner et al., 1984; Greenberger and Neil, 1992). Further research should consider that employment may alter parental perceptions of their child’s mental health, and therefore the child’s mental health status should be assessed by people other than the child’s parents, such as school teachers. Though, the different results found for boys and girls may be also attributable to their different mental health problems (Janitza et al., 2020) and health-related quality of life (O’Loughlin et al., 2023) trajectories throughout childhood and adolescence. Future studies should consider this, as well as examining the SDQ subscales, particularly to explore potential gender differences in the association between maternal employment and subsequent child mental health.

In our multivariable regression analysis, parental stress and anxiety at/before childbirth were associated with child mental health problems (Supplementary Tables S7–S10), similar to results of previous studies (Goodman et al., 2011; Achtergarde et al., 2015). Given that elevated levels of depressive symptoms were found in mothers who worked during the first 12 months postpartum (Brooks-Gunn et al., 2010), an indirect negative effect of maternal employment on child mental health, mediated by maternal mental health, is possible. Interestingly, the presence of an older sibling seemed to be associated with less mental health problems for both genders.

### Marginal part-time employment

For both gender there was a tendency that maternal marginal part-time employment at 1 year postpartum was associated with elevated levels of child mental health problems in children aged 4–6. Marginal part-time employment may be an indicator of a potential threat of a low income situation in Germany. This is because such jobs often have minimal requirements, a short and easy hiring process, and employment conditions in favor of the employer. This aligns with the family stress model, which postulates that poverty and economic pressure can lead to inter-parental conflict, which in turn can contribute to parent–child conflict and subsequent child problems (Masarik and Conger, 2017; Conger et al., 2010). This is supported by findings elsewhere that the transition into poverty within 11 years postpartum has a negative effect on child and maternal mental health (Wickham et al., 2017). Taken together with the positive findings regarding part-time employment 1 year postpartum found among boys, this suggests that the type of employment, and particularly the reasons for it may be important factors. This needs to be taken into account when interpreting the results of the trajectory classes indicating that boys whose mothers started labor work after 12 months postpartum have more mental health problems. In the long term, these mothers prevent the transition into poverty. Participation in the labor market while having young children is recommended from a long-term perspective. This conclusion is supported by another study analyzing maternal employment trajectories over a much longer period than ours (9 months, 3, 5 and 7 years postpartum) (Hope et al., 2014). The authors found that maternal employment was associated with fewer social and emotional difficulties at 7 years (Hope et al., 2014). We further want to posit that mixed findings were found for boys and no associations for girls, which might have stemmed from perception bias in parents. Our descriptive analysis revealed that mothers of boys in our cohort exhibited higher HADS scores during the period of exposure when compared to mothers of girls. It needs further elaboration if possible detrimental associations are attributable to a deficiency in an appropriate childcare situation for boys, support from partners of the mothers in the workforce, as well as from a lack of gender equality. Furthermore, limited policies to facilitate work-life balance may also be contributing factors. It is essential that public health policies give priority to the mental wellbeing of both parents and children.

### Limitations

If the quality of the paternal data had been equivalent to that of the maternal data in this birth cohort study, the same analytical approach would have been employed. Consequently, this analysis provides only a partial insight into the relationship between employment after childbirth and child mental health problems. To enhance the quality of future birth cohort studies, it is crucial to develop strategies that effectively engage both partners in the research process.

Our study did not assess data on child temperament which might have biased the results. Data on maternal occupation were not included in the analysis, as our focus was on working hours per week due to the high quality of the data in our cohort. However, we did adjust for maternal and paternal educational attainment, which is likely to be associated with occupation.

When considering the Bronfenbrenner’s Ecological Systems Theory (Bronfenbrenner et al., 1984; Bronfenbrenner, 1974), our study has some limitations: The mesosystem, an important component of a child’s development, was difficult to measure due to the observational nature of our study. Therefore, our results may be biased as the mesosystem might be associated with the mother’s labor force participation and the child’s mental health problems. In addition, the exosystem was only partially addressed in our study, as only maternal, and not paternal, employment was considered in our study. We considered the influence of the macrosystem to be similar for all participants in our study, living at the same time in a defined geographical region (south of Germany). However, the chronosystem was also only partially addressed: all children in our study can be considered to be in the same *normative* setting (e.g., not yet in school), but family circumstances can vary greatly and affect both the exposure and the outcome. For instance, a mother might have to stay home due to the death of her husband/wife or to care for older adult parents. We recognize that such “non-normative” events are associated with labor force participation. As these variables were not measured in our study and we only considered maternal and paternal mental health, our results are limited.

The interpretation of our results is limited by the sample size and the resulting lack of statistical power. In addition, we had a high proportion of families with high maternal educational attainment at baseline, which is representative of the local population, but families with low educational attainment and migrant backgrounds had higher loss to follow-up, especially during the first year of follow-up. Additionally, the mean maternal age at childbirth was slightly higher in the analysis sample than in the German population. Consequently, the analysis sample is biased towards relatively well-educated German families with no immigration background.

It is possible that those mothers who noticed a delay in their child’s socio-emotional development did not return to work (Hondralis and Kleinert, 2021), and therefore the reference group (no employment) may be confounded. Further, residual confounding cannot be ruled out, as mothers’ employment decisions depend on numerous factors (Hondralis and Kleinert, 2021) that also influence children’s mental health. For example, the results for boys (classes 2 and 3) could be explained by the fact that mothers in these classes are more likely to go to work due to a lack of family income. Based on the family stress model, economic pressure is associated with more inter-parental conflict, which in turn is associated with more parent–child problems, leading to more child problems (Masarik and Conger, 2017; Conger et al., 2010). Therefore, there may be other reasons than maternal employment for the results found.

Class 5 (“rapidly increasing – decreasing”) had the highest proportion of mothers having a second child 36 months postpartum [51.4% versus 24.0% in class 1 (“no or very low % of employment”), 8.6% in class 2 (“part-time after 1 year”), 5.3% in class 3 (“full time after 1 year”), and 6.4% in class 4 (“part-time after 2 years”)]. As this difference was considered in our analysis at each level of adjustment the results can be considered independent of this circumstance.

The relatively low number of mothers working full-time at 1 year postpartum in our sample has to be taken into account. This may be the reason for the statistically non-significant associations when comparing full-time vs. no employment at 1 year postpartum.

Due to the associative nature of the observational data and the statistical methodology used, this study does not allow causal conclusions to be drawn.

## Conclusion

Despite these limitations, we conclude that among girls no detrimental associations between maternal employment in the first 3 years postpartum and subsequent mental health at age 4, 5, and 6 were found. Mixed results were found for boys, depending on the measure used. Whether there are gender differences in the effects of maternal employment on children’s mental health problems, or whether this is an issue that is susceptible to parental perceptions (Bronfenbrenner et al., 1984; Greenberger and Neil, 1992) requires further investigation.

## Data Availability

The datasets presented in this article are not readily available because data will be made available upon reasonable request due to the ethical statement and data restrictions of this study. Requests to access the datasets should be directed to deborah.wernecke@uni-ulm.de.
