# Supplementary material for: Maternal employment patterns after childbirth and child mental health with 4–6 years of age
Source: Front Psychol. 2025 Aug 20;16:1580373. doi: 10.3389/fpsyg.2025.1580373 (PMC12405164; doi:10.3389/fpsyg.2025.1580373)
Supplement: Supplementary file 1 [file Supplementary_file_1.docx]

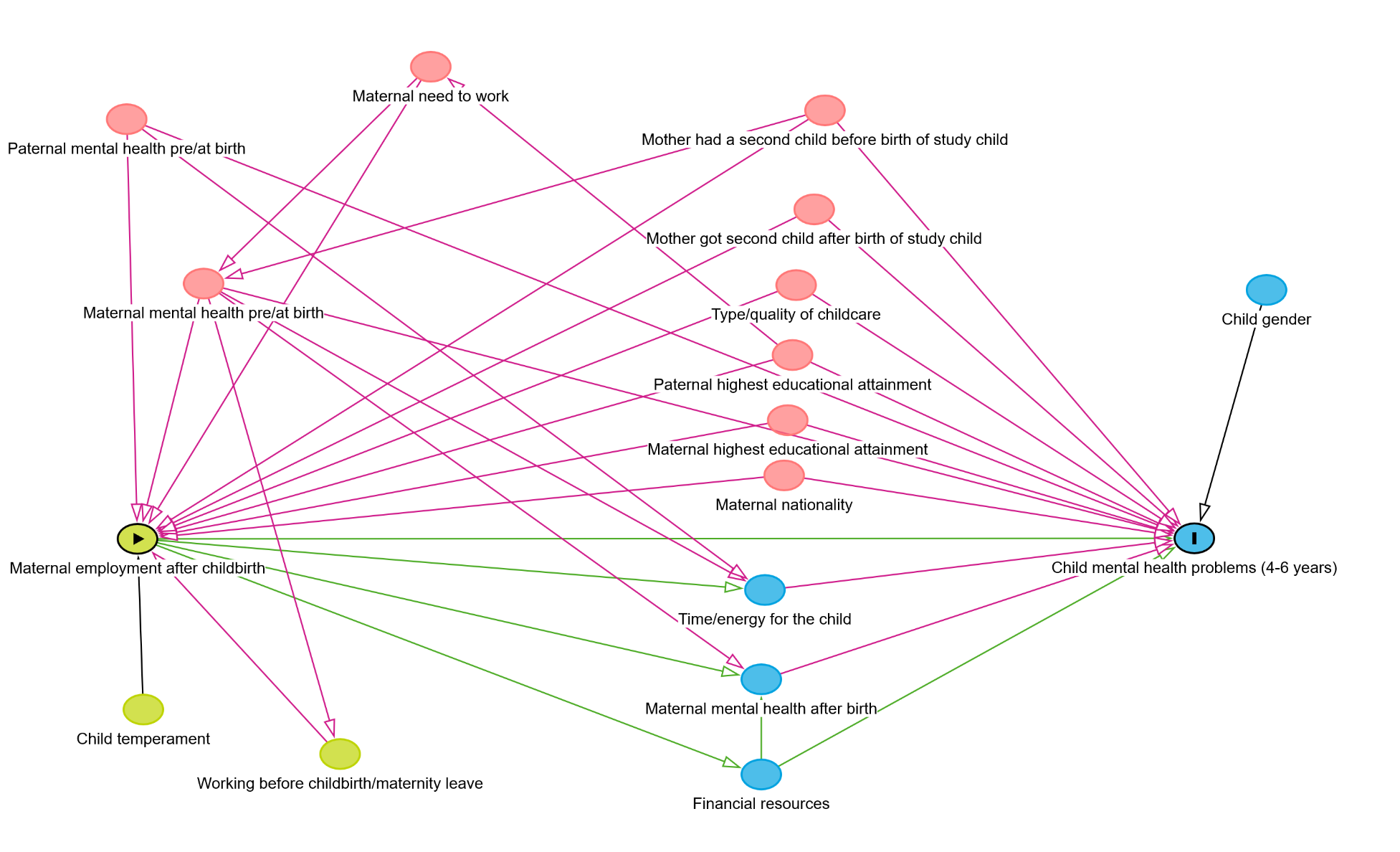


**Supplementary Figure S1.** Directed Acyclic Graph depicting the assumed associations between maternal emplyoment after childbirth and child mental health problems with 4-6 years of age, green variable=ancestor of exposure, blue variable= ancestor of outcome, red variable=ancestor or exposure and outcome, green path=causal path, red path=biasing path; Ulm SPATZ Health Study, Germany.

**Supplementary Methods**

*Type of childcare by time points after childbirth*

The type of childcare other than maternal care at 6 months, 1, 2, and 3 years was assessed with the following questions: *“When you think about a normal week, are there other people besides you who temporarily take care of the study child?”, “If yes, who are these people and how many hours per week do they provide care?”.* Based on the literature,^12^ four categories of care were distinguished: paternal care (in our sample, only one couple consisted of two mothers, second mother included here), center-based care, relative care (such as grandparents, siblings, or other relatives), and non-relative care (such as childminder, “nanny”, babysitter, or other non-relatives; excluding center-based care). We dichotomized these variables into ≥10 and <10 hours/week.^12^

*Statistical procedure for identification of trajectory classes*

We used a maximum of n=36 time points per individual (weekly working time per month after childbirth) based on questionnaire data collected at 6 months, 1, 2, and 3 years postpartum (T2-T5); (in sum 24,654 observations from 817 mothers.) To find appropriate “cut-points/time windows” for the general latent class mixed model estimating the trajectory classes we calculated %-change of working time to the previous time point (**Supplementary Figure S2**). The following time windows were identified and used to run the models: 0-6, 7-12, 13-24, and 25-36 months. The appropriate number of trajectory classes was determined by increasing it from 1 to 7 and subsequently staying with the number with the lowest Bayesian Information Criterion (BIC), given that the group size was ≥ 5% of all participants.


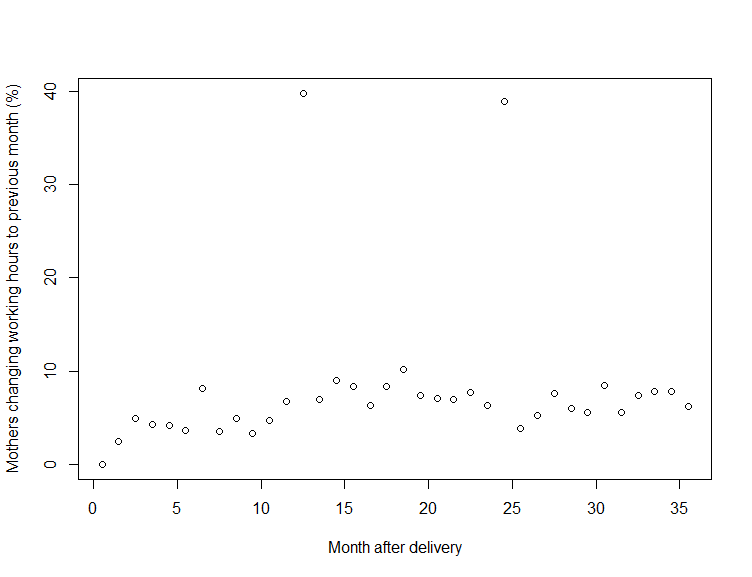


**Supplementary Figure S2.** Percentage of mothers in whom working time changed to previous months. Ulm SPATZ Health Study, Germany.

**Supplementary Figure S3.** Results of the identification of the appropriate number of trajectory classes. Ulm SPATZ Health Study, Germany.

**Supplementary Table S1.** Maternal weekly working time in months 1-36 after childbirth; data used for identification of trajectory classes; baseline: n=970 mothers. Ulm SPATZ Health Study, Germany.

| Months 1-12 after childbirth  (questionnaires at 6 months/1 year: n=768/726 mothers) | | | | | Months 13-24 after childbirth  (questionnaire at 2 years: n=694 mothers) | | | | | Months 25-36 after childbirth  (questionnaires at 3 years: n=625 mothers) | | | | |
| --- | --- | --- | --- | --- | --- | --- | --- | --- | --- | --- | --- | --- | --- | --- |
|  | | | Working time in hours/week | |  | | | Working time in hours/week | |  | | | Working time in hours/week | |
| months | N missing | N not employed | N working | Mean (SD),  Median (Min, Q1, Q3, Max) | months | N missing | N not employed | N working | Mean (SD),  Median (Min, Q1, Q3, Max) | months | N missing | N not employed | N working | Mean (SD),  Median (Min, Q1, Q3, Max) |
| 1 | 180 | 766 | 24 | 10.3 (12.8)  5.0 (1.0, 2.5, 10.0, 60.0) | 13 | 294 | 355 | 321 | 20.7 (12.7),  20.0 (1.0, 10.0, 30.0, 60.0) | 25 | 353 | 210 | 407 | 21.6 (11.0)  20.0 (2.0, 13.0, 30.0, 60.0) |
| 2 | 180 | 759 | 31 | 11.5 (12.2),  5.0, (1.0, 4.0, 16.0, 60.0) | 14 | 293 | 333 | 344 | 21.0 (12.5),  20.0 (1.0, 10.0, 30.0, 60.0) | 26 | 353 | 211 | 406 | 21.6 (10.8)  20.0 (2.0. 14.0, 30.0. 60.0) |
| 3 | 180 | 734 | 56 | 14.6 (13.4),  10.0 (1.0, 4.0, 22.5, 60.0) | 15 | 290 | 309 | 371 | 21.3 (12.3),  20.0 (1.0, 10.0, 30.0, 60.0) | 27 | 353 | 212 | 405 | 21.6 (10.9)  20.0 ( 2.0, 14.0, 30.0, 60.0) |
| 4 | 181 | 717 | 72 | 14.0 (11.9),  10.0 (1.0, 4.5, 20.0, 50.0) | 16 | 289 | 298 | 383 | 21.5 (11.9),  20.0 (1.0, 12.0, 30.0, 60.0) | 28 | 354 | 216 | 400 | 21.4 (10.8)  20.0 (2.0, 14.0, 30.0, 60.0) |
| 5 | 172 | 697 | 92 | 16.1 (13.6),  10.0 (1.0, 5.0, 25.5, 50.0) | 17 | 283 | 292 | 395 | 21.4 (11.8),  20.0, 1.0, 12.0, 30.0, 60.0) | 29 | 354 | 218 | 398 | 21.4 (11.0)  20.0 (2.0, 12.0, 30.0, 60.0) |
| 6 | 181 | 688 | 101 | 15.8 (13.3),  10.0 (1.0, 5.0, 25.0, 50.0) | 18 | 281 | 277 | 412 | 21.3 (11.6),  20.0 (1.0, 12.0, 30.0, 60.0) | 30 | 354 | 223 | 393 | 21.7 (11.1)  20.0 (2.0, 13.0, 30.0, 60.0) |
| 7 | 253 | 607 | 110 | 15.9 (14.0),  10.0 (1.0, 4.0, 28.0, 55.0) | 19 | 280 | 262 | 428 | 21.1 (11.6),  20.0 ( 1.0, 12.0, 30.0, 60.0) | 31 | 355 | 228 | 387 | 21.7 (11.1)  20.0 (2.0, 13.0, 30.0, 60.0) |
| 8 | 253 | 601 | 116 | 16.2 (14.7),  10.0 (1.0, 4.0, 29.0, 60.0) | 20 | 280 | 255 | 435 | 21.3 (11.5),  20.0 (1.0, 12.0, 30.0, 60.0) | 32 | 354 | 227 | 389 | 21.9 (11.1)  20.0 (2.0, 14.0, 30.0, 60.0) |
| 9 | 253 | 583 | 134 | 17.0 (14.7),  10.0 (1.0, 4.0, 30.0, 60.0) | 21 | 279 | 256 | 435 | 21.2 (11.3),  20.0 (1.0, 12.0, 30.0, 60.0) | 33 | 356 | 232 | 382 | 21.8 (11.1)  20.0 (2.0, 14.0, 30.0, 60.0) |
| 10 | 262 | 567 | 141 | 17.6 (14.6),  12.0 (1.0, 5.0, 30.0, 60.0) | 22 | 280 | 252 | 438 | 21.4 (11.4),  20.0 (1.0, 12.0, 30.0, 60.0) | 34 | 355 | 236 | 379 | 22.0 (10.9)  20.0 (2.0, 14.0, 30.0, 60.0) |
| 11 | 253 | 569 | 148 | 18.3 (14.6),  12.0 (1.0, 6.0, 30.0, 60.0) | 23 | 281 | 255 | 434 | 21.4 (11.3),  20.0 (1.0, 12.0, 30.0, 60.0) | 35 | 356 | 235 | 379 | 22.8 (11.0)  20.0 (2.0, 14.0, 30.0, 60.0) |
| 12 | 253 | 541 | 176 | 18.2 (13.8),  14.5 (1.0, 6.0, 30.0, 60.0) | 24 | 282 | 252 | 436 | 21.5 (11.1),  20.0 (1.0, 12.0, 30.0, 60.0) | 36 | 356 | 235 | 379 | 22.1 (10.9)  20.0 (2.0, 14.0, 30.0, 60.0) |

**Supplementary Table S2.** Type of child care used at 6 months, 1 year, 2 years, and 3 years after birth. Data from 1 year was used for adjustment in the main multivariable linear regression analysis. Ulm SPATZ Health Study, Germany.

|  |  | 6 months  N=794 children |  | 1 year  N=750 children |  | 2 years  N=719 children |  | 3 years  N=649 children |
| --- | --- | --- | --- | --- | --- | --- | --- | --- |
| Care of child by persons other than mother | N | N (%), or  Mean (SD) and Median (Min, Q1, Q3, Max) | N | N (%), or  Mean (SD) and Median (Min, Q1, Q3, Max) | N | N (%), or  Mean (SD) and Median (Min, Q1, Q3, Max) | N | N (%), or  Mean (SD) and Median (Min, Q1, Q3, Max) |
| Are there any other persons taking care of the child? |  |  |  |  |  |  |  |  |
| Yes | 794 | 631 (79.5) | 750 | 618 (82.4) | 719 | 643 (89.4) | 649 | 606 (93.4) |
| No |  | 131 (17.2) |  | 70 (9.3) |  | 27 (3.8) |  | 10 (1.5) |
| Missing |  | 32 (4.0) |  | 62 (8.3) |  | 49 (6.8) |  | 33 (5.1) |
| Paternal care | 794 | 615 (77.5) | 750 | 586 (78.1) | 719 | 554 (77.1) | 649 | 501 (77.2) |
| Hours/week | 561 | 12.8 (13.0)  10.0 (1.0, 5.0, 15.0, 84.0) | 528 | 14.8 (14.0)  10.0 (1.0, 5.0, 20.0, 90.0) | 499 | 14.9 (13.1)  10.0 (1.0, 6.0, 20.0, 90.0) | 440 | 16.2 (14.9)  10.0 (1.0, 6.0, 20.0, 84.0) |
| Paternal care ≥10h/week | 794 | 290 (36.5) | 750 | 329 (43.9) | 719 | 315 (43.8) | 649 | 280 (43.1) |
| Center-based care | 794 | 8 (1.0) | 750 | 90 (12.0) | 719 | 321 (44.7) | 649 | 463 (71.3) |
| Hours/week | 6 | 30.2 (9.2)  30.0 (16.0, 25.0, 40.0, 40.0) | 84 | 22.5 (10.9)  25.0 (2.0, 15.0, 30.0, 47.0) | 318 | 28.4 (8.9)  30.0 (4.0, 24.0, 35.0, 56.0) | 458 | 26.2 (9.3)  5.5 (2.0, 20.0, 33.0, 50.0) |
| Center-based care ≥10h/week | 794 | 6 (0.8) | 750 | 75 (10.0) | 719 | 311 (43.3) |  | 434 (66.9) |
| Relative care^a^ | 794 | 357 (45.0) | 750 | 412 (54.9) | 719 | 411 (57.2) | 649 | 380 (58.6) |
| Hours/week | 333 | 5.8 (7.4)  3.0 (0.5, 2.0, 7.0, 60.0) | 397 | 7.4 (7.7)  5.0 (0.5, 3.0, 10.0, 60.0) | 395 | 8.3 (7.7)  6.0 (1.0, 3.0, 10.0, 50.0) | 362 | 8.1 (7.5)  6.0 (1.0, 3.0, 10.0, 60.0) |
| Relative care ≥10h/week | 794 | 63 (7.9) | 750 | 107 (14.3) | 719 | 128 (17.8) | 649 | 116 (17.9) |
| Non-relative care^b^ | 794 | 38 (4.8) | 750 | 65 (8.7) | 719 | 93 (12.9) | 649 | 75 (11.6) |
| Hours/week | 38 | 6.5 (9.9)  2.5 (1.0, 2.0, 5.0, 50.0) | 58 | 9.7 (10.6)  5.0 (1.0, 2.0, 15.0, 40.0) | 86 | 10.3 (11.0)  5.0 (1.0, 2.0, 15.0, 48.0) | 68 | 7.3 (8.6)  3.0 (1.0, 2.0, 9.0, 40.0) |
| Non-relative care ≥10h/week | 794 | 7 (0.9) | 750 | 20 (2.7) | 719 | 35 (4.9) | 649 | 17 (2.6) |

^a^such as grandparents, siblings, relatives.

^b^such as childminder, nanny, babysitter, or other persons; excludes center-based care. h: hours.

**Supplementary Table S3**. Mental health problems of children aged 4, 5, and 6 indexed by the total SDQ-score, overall and by maternal employment after childbirth. (n=5 trajectory classes, and working status at 12 months after childbirth). Ulm SPATZ Health Study, Germany.

|  | Outcome total SDQ-score by child age | | | | | | | | |
| --- | --- | --- | --- | --- | --- | --- | --- | --- | --- |
| Exposures | 4 years | | | 5 years | | | 6 years | | |
|  | n boys, n girls | Mean (SD) | | n boys, n girls | Mean (SD) | | n boys, n girls | Mean (SD) | |
|  |  | boys | girls |  | boys | girls |  | boys | girls |
| Total SDQ-score | 279, 278 | 8.0 (4.6) | 7.2 (4.7) | 247, 246 | 7.9 (4.7) | 6.4 (4.5) | 208, 210 | 7.3 (5.1) | 6.1 (4.3) |
| Median  (Min, Q1, Q3, Max) |  | 7.0  (0.0, 5.0, 11.0, 25.0) | 6.0  (0.0, 4.0, 10.0. 30.0) |  | 7.0  (0.0, 4.0, 11.0, 26.0) | 6.0  (0.0, 3.0, 9.0, 29.5) |  | 7.0  (0.0, 3.0, 10.0, 31.0) | 5.0  (0.0, 3.0, 9.0, 26.0) |
| Trajectory classes |  |  |  |  |  |  |  |  |  |
| Class 1 | 121, 128 | 8.4 (4.9) | 7.4 (4.8) | 104, 109 | 8.0 (5.2) | 6.4 (4.1) | 84, 91 | 7.0 (6.1) | 6.6 (4.0) |
| Class 2 | 69, 71 | 7.5 (4.0) | 6.7 (4.5) | 65, 62 | 7.6 (4.2) | 6.0 (5.3) | 56, 53 | 7.5 (4.4) | 6.1 (4.7) |
| Class 3 | 42, 38 | 8.5 (4.4) | 7.0 (4.9) | 36, 36 | 8.3 (4.6) | 7.3 (5.3) | 31, 36 | 8.2 (4.4) | 5.6 (4.8) |
| Class 4 | 25, 25 | 8.1 (5.0) | 7.2 (5.7) | 20, 23 | 7.4 (3.6) | 5.8 (3.4) | 19, 17 | 6.9 (4.3) | 6.1 (3.8) |
| Class 5 | 22, 16 | 7.0 (4.2) | 7.2 (3.0) | 22, 16 | 8.0 (4.8) | 6.2 (3.2) | 18, 13 | 7.0 (3.7) | 4.4 (2.8) |
| Maternal employment at 12 m. after childbirth |  |  |  |  |  |  |  |  |  |
| Full-time (≥ 35h/week) | 20, 21 | 8.1 (4.4) | 6.9 (4.3) | 18, 21 | 7.9 (5.4) | 6.0 (3.8) | 17, 17 | 8.3 (3.8) | 4.5 (3.9) |
| Part-time (35h/week) | 33, 53 | 6.9 ( 3.9) | 6.5 (4.2) | 31, 47 | 7.2 (3.8) | 6.0 (4.4) | 25, 41 | 7.1 (4.7) | 5.6 (4.4) |
| Apprenticeship^a^ | 1, 1 | 10.0 (-) | 6.0 (-) | 1, 1 | 10.0 (-) | 6.0 (-) | 1, 1 | 11.0 (-) | 11.0 (-) |
| Marginal part-time employment | 18, 30 | 7.1 (3.6) | 7.6 (5.0) | 16, 24 | 7.7 (5.1) | 7.9 (6.1) | 13, 19 | 6.8 (4.0) | 7.3 (5.9) |
| Not employed | 194, 157 | 8.4 (4.8) | 7.3 (4.7) | 171, 142 | 8.1 (4.8) | 6.3 (4.3) | 144, 124 | 7.3 (5.4) | 6.3 (3.9) |
| Missing | 13, 16 | 6.9 (4.5) | 8.1 (6.5) | 10, 11 | 7.8 (4.8) | 5.6 (5.5) | 8, 8 | 7.4 (6.3) | 5.7 (4.3) |

SDQ-score: lower scores indicate fewer social and emotional difficulties. Class 1: “no or very low % of employment”, class 2: “part-time after 1 year”, class 3: “full time after 1 year”, class 4: “part-time after 2 years”, class 5: “rapidly increasing – decreasing”. Please see also Figure 1 ^a^for statistical models considered as full-time work. h: hours; m: months; Q1: first quartile; Q3: third quartile.

**Supplementary Table S4**. Health-related quality of life of children aged 4, 5, and 6 indexed by the total KINDL-score, overall and by maternal employment after childbirth (n=5 trajectory classes, and working status 12 months after childbirth). Ulm SPATZ Health Study, Germany.

|  | Outcome total KINDL-score by child age | | | | | | | | |
| --- | --- | --- | --- | --- | --- | --- | --- | --- | --- |
| Exposures | 4 years | | | 5 years | | | 6 years | | |
|  | n boys, n girls | Mean (SD) | | n boys, n girls | Mean (SD) | | n boys, n girls | Mean (SD) | |
|  |  | boys | girls |  | boys | girls |  | boys | girls |
| Total SDQ-score | 272, 273 | 79.8 (7.5) | 80.9 (8.1) | 241, 238 | 79.8 (8.2) | 81.4 (7.7) | 185, 180 | 79.7 (8.0) | 80.8 (7.7) |
| Median  (Min, Q1, Q3, Max) |  | 80.2 (56.3, 75.5, 84.4, 95.8) | 82.3 (54.2, 76.0, 86.5, 95.8) |  | 80.2 (40.6, 76.0, 84.4, 96.9) | 81.3 (50.0, 76.0, 87.5, 95.8) |  | 80.2 (45.8, 75.0, 84.4, 95.8) | 80.7 (55.2, 76.0, 86.5, 95.8) |
| Trajectory classes |  |  |  |  |  |  |  |  |  |
| Class 1 | 117, 124 | 79.5 (8.2) | 79.9 (8.9) | 98, 104 | 79.2 (8.8) | 80.8 (7.4) | 75, 79 | 79.2 (9.4) | 81.0 (6.9) |
| Class 2 | 70, 71 | 79.6 (7.1) | 82.0 (7.6) | 64, 59 | 80.8 (7.1) | 82.1 (8.4) | 50, 46 | 79.4 (7.4) | 80.8 (8.7) |
| Class 3 | 40, 38 | 81.5 (6.7) | 81.6 (7.2) | 37, 36 | 81.5 (7.7) | 80.4 (8.1) | 23, 31 | 78.8 (6.3) | 79.5 (8.8) |
| Class 4 | 24, 24 | 78.9 (7.2) | 80.9 (7.1) | 20, 23 | 78.3 (7.2) | 83.5 (7.0) | 19, 14 | 81.0 (5.9) | 81.0 (6.6) |
| Class 5 | 21, 16 | 80.4 (6.9) | 82.4 (6.1) | 22, 16 | 78.0 (9.6) | 82.1 (5.8) | 18, 10 | 82.4 (6.7) | 83.5 (7.4) |
| Maternal employment at 12 m. after childbirth |  |  |  |  |  |  |  |  |  |
| Full-time (≥ 35h/week) | 21, 22 | 79.2 (7.6) | 82.2 (5.9) | 19, 21 | 78.2 (9.1) | 82.4 (6.9) | 14, 13 | 81.9 (7.8) | 80.4 (7.8) |
| Part-time (35h/week) | 34, 52 | 80.2 (6.3) | 82.6 (7.7) | 31, 47 | 80.4 (6.6) | 82.3 (8.5) | 22, 35 | 80.8 (6.7) | 82.7 (8.7) |
| Apprenticeship^a^ | 1, 1 | 75.0 (-) | 84.4 (-) | 1, 1 | 71.9 (-) | 92.7 (-) | 1, 1 | 71.9 (-) | 55.2 (-) |
| Marginal part-time  employment | 18, 28 | 79.2 (8.6) | 80.5 (8.2) | 14, 23 | 77.8 (7.8) | 80.1 (9.4) | 12, 15 | 76.1 (7.7) | 77.8 (8.0) |
| Not employed | 186, 153 | 80.1 (7.8) | 80.6 (8.3) | 166, 135 | 80.0 (8.4) | 81.0 (7.1) | 130, 109 | 80.0 (8.0) | 80.8 (6.9) |
| Missing | 12, 17 | 77.2 (5.4) | 78.4 (9.1) | 10, 11 | 82.1 (8.0) | 82.0 (7.6) | 6, 7 | 73.4 (11.0) | 83.3 (6.0) |

KINDL-score: higher scores indicate better health-related quality of life. Class 1: “no or very low % of employment”, class 2: “part-time after 1 year”, class 3: “full time after 1 year”, class 4: “part-time after 2 years”, class 5: “rapidly increasing – decreasing”. Please see also Figure 1 ^a^for statistical models considered as full-time work. h: hours; m: months; Q1: first quartile; Q3: third quartile.

Summary Tables S3, S4: Comparing the descriptive results by trajectory class, the highest SDQ scores for boys were found in trajectory class 3 at 4, 5, and 6 years. For girls, the highest scores were found in class 1 (4, and 6 years) and class 3 (5 years). Comparing descriptive results by maternal employment status, the highest health-related quality of life, and the fewest emotional and social difficulties with 4, and 5 years of age were found among maternal part-time employment. In contrast, the lowest health-related quality of life with 4, 5, and 6 years was found among maternal marginal-part time employment. Girls in this group also showed the highest SDQ-scores.

**Supplementary Table S5**. Associations between maternal employment after childbirth (n=5 trajectory classes, and working status 12 months after childbirth) and mental health problems of children aged 4, 5 and 6 indexed by the total SDQ-score (results of **crude linear regression** models). Ulm SPATZ Health Study, Germany.

|  |  | Difference in means (95% CI) in total SDQ-scores by child age | | | | | | |  |
| --- | --- | --- | --- | --- | --- | --- | --- | --- | --- |
|  | n | 4 years | p | n | 5 years | p | n | 6 years | p |
| Trajectory classes |  |  |  |  |  |  |  |  |  |
| BOYS | 279 |  |  | 247 |  |  | 208 |  |  |
| Class 1 |  | ref. |  |  | ref. |  |  | ref. |  |
| Class 2 |  | -0.83 (-2.11, 0.45) | 0.21 |  | 0.41 (-1.84, 1.01) | 0.57 |  | 0.48 (-1.26, 2.22) | 0.59 |
| Class 3 |  | 0.14 (-1.43, 1.70) | 0.86 |  | 0.27 (-1.51, 2.05) | 0.77 |  | 1.18 (-0.84, 3.19) | 0.25 |
| Class 4 |  | -0.28 (-2.39, 1.83) | 0.79 |  | -0.63 (-2.48, 1.22) | 0.50 |  | -0.19 (-2.48, 2.10) | 0.87 |
| Class 5 |  | -1.36 (-3.29, 0.56) | 0.17 |  | -0.03 (-2.24, 2.18) | 0.98 |  | -0.04 (-2.15, 2.07) | 0.97 |
| GIRLS | 278 |  |  | 246 |  |  | 210 |  |  |
| Class 1 |  | ref. |  |  | ref. |  |  | ref. |  |
| Class 2 |  | -0.73 (-2.05, 0.59) | 0.28 |  | -0.36 (-1.88, 1.16) | 0.65 |  | -0.48 (-1.98, 1.02) | 0.53 |
| Class 3 |  | -0.42 (-2.16, 1.31) | 0.63 |  | 0.91 (-0.97, 2.79) | 0.34 |  | -1.00 (-2.76, 0.76) | 0.26 |
| Class 4 |  | -0.20 (-2.55, 2.14) | 0.87 |  | -0.56 (-2.11, 0.99) | 0.48 |  | -0.48 (-2.40, 1.43) | 0.62 |
| Class 5 |  | -0.26 (-1.92, 1.41) | 0.76 |  | -0.20 (-1.90, 1.50) | 0.82 |  | **-2.17 (-3.84, -0.51)** | **0.01** |
| Employment at 12 m. |  |  |  |  |  |  |  |  |  |
| BOYS | 266 |  |  | 237 |  |  | 200 |  |  |
| Not employed |  | ref. |  |  | ref. |  |  | ref. |  |
| Marginal part-time |  | -1.29 (-3.02, 0.44) | 0.14 |  | -0.37 (-2.90, 2.17) | 0.78 |  | -0.42 (-2.70, 1.84) | 0.71 |
| Part-time |  | -1.46 (-2.92, 0.003) | 0.05 |  | -0.85 (-2.35, 0.65) | 0.27 |  | -0.20 (-2.21, 1.81) | 0.85 |
| Full-time |  | -0.19 (-2.11, 1.73) | 0.84 |  | -0.05 (-2.48, 2.37) | 0.97 |  | 1.17 (-0.72, 3.07) | 0.22 |
| GIRLS | 262 |  |  | 235 |  |  | 202 |  |  |
| Not employed |  | ref. |  |  | ref. |  |  | ref. |  |
| Marginal part-time |  | 0.29 (-1.62, 2.20) | 0.77 |  | 1.56 (-0.92, 4.05) | 0.22 |  | 1.00 (-1.68, 3.67) | 0.46 |
| Part-time |  | -0.79 (-2.13, 0.54) | 0.24 |  | -0.36 (-1.78, 1.06) | 0.62 |  | -0.63 (-2.14, 0.88) | 0.41 |
| Full-time |  | -0.42 (-2.27, 1.44) | 0.66 |  | -0.34 (-2.01, 1.33) | 0.69 |  | -1.37 (-3.33, 0.58) | 0.17 |

SDQ-scores: lower scores indicate fewer social and emotional difficulties. Class 1: “no or very low % of employment”, class 2: “part-time after 1 year”, class 3: “full time after 1 year”, class 4: “part-time after 2 years”, class 5: “rapidly increasing – decreasing”. Please see also Figure 1 Full-time: ≥35h/week (includes n=2 mothers doing an apprenticeship); part-time: <35h/week; marginal part-time: employment such as mini-jobs or irregular jobs with very few working hours; CI: Confidence intervall; m: months;

**Supplementary Table S6**. Associations between maternal employment after childbirth (n=5 trajectory classes, and working status 12 months after childbirth) and health-related quality of life of children aged 4, 5 and 6 indexed with the KINDL-score (results of crude linear regression models). Ulm SPATZ Health Study, Germany.

|  |  | Difference in means (95% CI) in total KINDL-scores by child age | | | | | | |  |
| --- | --- | --- | --- | --- | --- | --- | --- | --- | --- |
|  | n | 4 years | p | n | 5 years | p | n | 6 years | p |
| Trajectory classes |  |  |  |  |  |  |  |  |  |
| BOYS | 272 |  |  | 241 |  |  | 185 |  |  |
| Class 1 |  | ref. |  |  | ref. |  |  | ref. |  |
| Class 2 |  | 0.01 (-2.20, 2.23) | 0.99 |  | 1.57 (-0.87, 4.01) | 0.21 |  | 0.20 (-2.74, 3.14) | 0.89 |
| Class 3 |  | 1.93 (-0.59, 4.46) | 0.13 |  | 2.27 (-0.74, 5.27) | 0.14 |  | -0.39 (-3.68, 2.90) | 0.82 |
| Class 4 |  | -0.66 (-3.85, 2.54) | 0.69 |  | -0.94 (-4.48, 2.60) | 0.60 |  | 1.78 (-1.57, 5.13) | 0.30 |
| Class 5 |  | 0.86 (-2.39, 4.11) | 0.61 |  | -1.27 (-5.54, 3.00) | 0.56 |  | 3.21 (-0.47, 6.89) | 0.09 |
| GIRLS | 273 |  |  | 238 |  |  | 180 |  |  |
| Class 1 |  | ref. |  |  | ref. |  |  | ref. |  |
| Class 2 |  | 2.09 (-0.25, 4.43) | 0.08 |  | 1.28 (-1.27, 3.83) | 0.33 |  | -0.25 (-3.15, 2.65) | 0.87 |
| Class 3 |  | 1.69 (-1.07, 4.45) | 0.23 |  | -0.42 (-3.40, 2.57) | 0.78 |  | -1.46 (-4.85, 1.93) | 0.40 |
| Class 4 |  | 0.91 (-2.26, 4.09) | 0.57 |  | 2.65 (-0.50, 5.80) | 0.10 |  | -0.05 (-3.71, 3.61) | 0.98 |
| Class 5 |  | 2.48 (-0.82, 5.78) | 0.14 |  | 1.28 (-1.84, 4.39) | 0.42 |  | 2.54 (-2.06, 7.14) | 0.28 |
| Employment at 12 m. |  |  |  |  |  |  |  |  |  |
| BOYS | 260 |  |  | 231 |  |  | 179 |  |  |
| Not employed |  | ref. |  |  | ref. |  |  | ref. |  |
| Marginal part-time |  | -0.93 (-4.92, 3.07) | 0.65 |  | -2.21 (-6.36, 1.94) | 0.30 |  | -3.83 (-8.20, 0.55) | 0.09 |
| Part-time |  | 0.08 (-2.28, 2.44) | 0.95 |  | 0.47 (-2.14, 3.07) | 0.72 |  | 0.80 (-2.27, 3.88) | 0.61 |
| Full-time |  | -1.12 (-4.38, 2.14) | 0.50 |  | -2.10 (-6.13, 1.93) | 0.31 |  | 1.29 (-2.81, 5.40) | 0.54 |
| GIRLS | 256 |  |  | 227 |  |  | 173 |  |  |
| Not employed |  | ref. |  |  | ref. |  |  | ref. |  |
| Marginal part-time |  | -0.12 (-3.38, 3.14) | 0.94 |  | -0.98 (-4.93, 2.98) | 0.63 |  | -2.98 (-7.10, 1.14) | 0.16 |
| Part-time |  | 1.98 (-0.47, 4.44) | 0.11 |  | 1.26 (-1.42, 3.95) | 0.36 |  | 1.92 (-1.20, 5.03) | 0.23 |
| Full-time |  | 1.71 (-0.94, 4.37) | 0.21 |  | 1.79 (-1.34, 4.93) | 0.26 |  | -2.12 (-7.36, 3.13) | 0.43 |

KINDL-scores: higher scores indicate better health-related quality of life; Class 1: “no or very low % of employment”, class 2: “part-time after 1 year”, class 3: “full time after 1 year”, class 4: “part-time after 2 years”, class 5: “rapidly increasing – decreasing”. Please see also Figure 1 Full-time: ≥35h/week (includes n=2 mothers doing an apprenticeship); Part-time: <35h/week; marginal part-time: employment such as mini-jobs or irregular jobs with very few working hours. CI: Confidence intervall; m: months.

**Supplementary Table S7.** Adjustment variables in linear regression models estimating the association betwenn maternal employment after childbirth (n=5 trajectory classes) and mental health problems of children aged 4, 5, and 6 indexed with the total SDQ-score by gender. Ulm SPATZ Health Study, Germany.

|  |  | Difference in means (95% CI) in total SDQ-scores by child age | | | | | | | |
| --- | --- | --- | --- | --- | --- | --- | --- | --- | --- |
|  |  | 4 years | p |  | 5 years | p |  | 6 years | p |
| Exposure trajectory classes |  |  |  |  |  |  |  |  |  |
| BOYS |  |  |  |  |  |  |  |  |  |
| Had second child |  | **-1.63 (-2.98, -0.29)** | **0.02** |  | **-0.64 (-2.17, 0.89)** | **0.41** |  | 0.09 (-1.65, 1.83) | 0.92 |
| Got second child |  | **1.84 (0.01, 3.67)** | **0.049** |  | **2.93 (0.70, 5.16)** | **0.01** |  | **3.95 (1.24, 6.65)** | **0.004** |
| Regular childcare other than mother at 12 months  (≥10h/week vs <10h/week) |  |  |  |  |  |  |  |  |  |
| Partner |  | -0.20 (-1.41, 1.02) | 0.75 |  | 0.67 (-0.73, 2.06) | 0.35 |  | 0.84 (-0.64, 2.32) | 0.27 |
| Center-based care |  | -1.31 (-3.45, 0.82) | 0.23 |  | **-2.51 (-4.37, -0.64)** | **0.008** |  | -0.92 (-3.35, 1.50) | 0.46 |
| Relative care |  | 0.68 (-1.12, 2.48) | 0.46 |  | -0.46 (-2.38, 1.46) | 0.64 |  | 0.78 (-1.21, 2.77) | 0.44 |
| Non-relative care |  | 0.76 (-2.92, 4.44) | 0.69 |  | **4.78 (1.75, 7.81)** | **0.002** |  | 0.06 (-4.69, 4.80) | 0.98 |
| Anxiety and depression symptoms pre/at birth |  |  |  |  |  |  |  |  |  |
| Maternal HADS, per 1 SD |  | 0.32 (-0.30, 0.94) | 0.32 |  | 0.56 (-0.20, 1.33) | 0.15 |  | 0.61 (-0.34, 1.55) | 0.21 |
| Maternal TICS-t, per 1 SD |  | **1.13 (0.49, 1.77)** | **<0.001** |  | **1.19 (0.52, 1.86)** | **<0.001** |  | **0.71 (-0.07, 1.50)** | **0.08** |
| Paternal TICS-t, per 1 SD |  | **0.88 (0.26, 1.50)** | **0.006** |  | **0.70 (0.004, 1.39)** | **0.049** |  | **0.72 (-0.10, 1.53)** | **0.09** |
| Maternal education  (<12 yrs vs ≥12 yrs) |  | -0.18 (-1.64, 1.29) | 0.81 |  | **1.64 (0.08, 3.20)** | **0.04** |  | 1.19 (-0.38, 2.76) | 0.14 |
| Paternal education  (<12 yrs vs ≥12 yrs) |  | 0.79 (-0.54, 2.13) | 0.25 |  | -0.18 (-1.48, 1.11) | 0.78 |  | -0.73 (-2.18, 0.71) | 0.32 |
| Maternal nationality (German vs non-German) |  | 0.95 (-1.00, 2.89 | 0.34 |  | -1.36 (-4.56, 1.85) | 0.41 |  | -0.07 (-2.83, 2.68) | 0.96 |
| GIRLS |  |  |  |  |  |  |  |  |  |
| Had second child |  | -1.22 (-2.64, 0.20) | 0.09 |  | **-1.96 (-3.37, -0.56)** | **0.006** |  | **-2.10 (-3.50, -0.71)** | **0.003** |
| Got second child |  | 0.05 (-1.77, 1.87) | 0.96 |  | -0.38 (-2.13, 1.37) | 0.67 |  | **-1.97 (-3.87, -0.08)** | **0.04** |
| Regular childcare other than mother at 12 months  (≥10h/week vs <10h/week) |  |  |  |  |  |  |  |  |  |
| Partner |  | -0.01 (-1.43, 1.40) | 0.98 |  | 0.84 (-0.65, 2.33) | 0.27 |  | 1.26 (-0.13, 2.65) | 0.08 |
| Center-based care |  | 0.82 (-1.50, 3.14) | 0.49 |  | -0.31 (-2.53, 1.92) | 0.79 |  | 0.74 (-1.73, 3.21) | 0.56 |
| Relative care |  | -0.58 (-2.11, 0.95) | 0.46 |  | -0.64 (-2.08, 0.80) | 0.39 |  | **-1.46 (-2.91, -0.005)** | **0.049** |
| Non-relative care |  | -0.25 (-4.19, 3.68) | 0.90 |  | 0.43 (-1.83, 2.69) | 0.71 |  | 1.80 (-2.23, 5.82) | 0.38 |
| Anxiety and depression symptoms pre/at birth |  |  |  |  |  |  |  |  |  |
| Maternal HADS, per 1 SD |  | 0.61 (-0.38, 1.59) | 0.23 |  | 0.49 (-0.36, 1.34) | 0.26 |  | 0.33 (-0.58, 1.24) | 0.48 |
| Maternal TICS-t, per 1 SD |  | 0.47 (-0.43, 1.37) | 0.30 |  | **0.69 (0.004, 1.38)** | **0.049** |  | 0.69 (-0.08, 1.46) | 0.08 |
| Paternal TICS-t, per 1 SD |  | 0.39 (-0.39, 1.16) | 0.33 |  | 0.13 (-0.68, 0.93) | 0.76 |  | **0.80 (0.17, 1.42)** | **0.01** |
| Maternal education  (<12 yrs vs ≥12 yrs) |  | 0.21 (-1.38, 1.79) | 0.80 |  | 0.32 (-1.89, 2.53) | 0.78 |  | 0.37 (-1.66, 2.41) | 0.72 |
| Paternal education  (<12 yrs vs ≥12 yrs) |  | 0.57 (-1.33, 2.48) | 0.55 |  | 0.90 (-1.51, 3.31) | 0.47 |  | 0.12 (-2.07, 2.31) | 0.91 |
| Maternal nationality (German vs non-German) |  | -0.14 (-4.04, 3.76) | 0.94 |  | 0.31 (-3.30, 3.91) | 0.87 |  | 0.44 (-2.35, 3.22) | 0.76 |

SDQ-scores: lower scores indicate fewer social and emotional difficulties CI: confidence intervall; h: hours; vs: versus; w: week; yrs: years.

**Supplementary Table S8.** Adjustment variables in linear regression models estimating the association betwenn maternal employment after childbirth (employment status 12 months after childbirth) and mental health problems of children aged 4, 5, and 6 indexed with the total SDQ-score by gender. Ulm SPATZ Health Study, Germany.

|  |  | Difference in means (95% CI) in total SDQ-scores by child age | | | | | | | |
| --- | --- | --- | --- | --- | --- | --- | --- | --- | --- |
|  |  | 4 years | p |  | 5 years | p |  | 6 years | p |
| Exposure employment status 12 months after childbirth |  |  |  |  |  |  |  |  |  |
| BOYS |  |  |  |  |  |  |  |  |  |
| Had second child |  | **-1.49 (-2.85, -0.13)** | **0.032** |  | -0.69 (-2.28, 0.91) | 0.40 |  | -0.05 (-1.85, 1.75) | 0.96 |
| Got second child |  | 1.48 (-0.27, 3.23) | 0.10 |  | **2.37 (0.18, 4.57)** | **0.03** |  | **3.25 (0.62, 5.88)** | **0.02** |
| Regular childcare other than mother at 12 months  (≥10h/w. vs <10h/w.) |  |  |  |  |  |  |  |  |  |
| Partner |  | -0.16 (-1.41, 1.08) | 0.79 |  | 0.64 (-0.75, 2.03) | 0.37 |  | 1.10 (-0.49, 2.70) | 0.17 |
| Center-based care |  | -0.36 (-1.92, 1.20) | 0.65 |  | -1.43 (-3.38, 0.51) | 0.15 |  | -0.52 (-3.19, 2.15) | 0.70 |
| Relative care |  | 1.34 (-0.51, 3.19) | 0.15 |  | 0.18 (-2.17, 2.52) | 0.88 |  | 0.90 (-1.27, 3.07) | 0.42 |
| Non-relative care |  | 2.52 (-0.89, 5.93) | 0.15 |  | **5.76 (2.09, 9.43)** | **0.002** |  | -0.04 (-4.84, 4.77) | 0.99 |
| Anxiety and depression symptoms pre/at birth |  |  |  |  |  |  |  |  |  |
| Maternal HADS, per 1 SD |  | 0.27 (-0.36, 0.90) | 0.40 |  | 0.36 (-0.45, 1.17) | 0.38 |  | 0.54 (-0.49, 1.57) | 0.30 |
| Maternal TICS-t, per 1 SD |  | **1.21 (0.56, 1.85)** | **<0.001** |  | **1.13 (0.41, 1.84)** | **0.002** |  | **0.83 (0.05, 1.60)** | **0.04** |
| Paternal TICS-t, per 1 SD |  | **0.79 (0.16, 1.43)** | **0.015** |  | 0.56 (-0.18, 1.30) | 0.14 |  | 0.60 (-0.28, 1.48) | 0.18 |
| Maternal education  (<12 yrs vs ≥12 yrs) |  | -0.10 (-1.49, 1.29) | 0.89 |  | **1.68 (0.10, 3.26)** | **0.04** |  | 1.33 (-0.30, 2.96) | 0.11 |
| Paternal education  (<12 yrs vs ≥12 yrs) |  | 1.01 (-0.23, 2.26) | 0.11 |  | -0.19 (-1.54, 1.15) | 0.78 |  | -0.39 (-1.85, 1.07) | 0.60 |
| Maternal nationality (German vs non-German) |  | 0.34 (-1.67, 2.35) | 0.74 |  | -1.57 (-5.02, 1.89) | 0.37 |  | 0.17 (-3.20, 3.54) | 0.92 |
| GIRLS |  |  |  |  |  |  |  |  |  |
| Had second child |  | -1.19 (-2.68, 0.31) | 0.12 |  | **-2.00 (-3.57, -0.44)** | **0.01** |  | **-1.98 (-3.44, -0.53)** | **0.008** |
| Got second child |  | -0.15 (-1.82, 1.52) | 0.86 |  | -0.87 (-2.36, 0.62) | 0.25 |  | -1.68 (-3.52, 0.17) | 0.07 |
| Regular childcare other than mother at 12 months  (≥10h/w. vs <10h/w.) |  |  |  |  |  |  |  |  |  |
| Partner |  | -0.01 (-1.38 1.35) | 0.99 |  | 0.65 (-0.74, 2.05) | 0.36 |  | 1.13 (-0.21, 2.48) | 0.10 |
| Center-based care |  | 0.70 (-1.93, 3.33) | 0.60 |  | -0.35 (-2.89, 2.19) | 0.79 |  | 0.53 (-2.52, 3.58) | 0.73 |
| Relative care |  | -0.81 (-2.56, 0.94) | 0.36 |  | -1.18 (-2.70, 0.34) | 0.13 |  | **-1.87 (-3.42, -0.31)** | **0.02** |
| Non-relative care |  | -0.53 (-4.33, 3.26) | 0.78 |  | -0.99 (-3.58, 1.60) | 0.45 |  | -0.94 (-4.20, 2.32) | 0.57 |
| Anxiety and depression symptoms pre/at birth |  |  |  |  |  |  |  |  |  |
| Maternal HADS, per 1 SD |  | 0.50 (-0.53, 1.53) | 0.34 |  | 0.27 (-0.55, 1.08) | 0.52 |  | 0.14 (-0.72, 1.00) | 0.75 |
| Maternal TICS-t, per 1 SD |  | 0.52 (-0.47, 1.50) | 0.30 |  | **0.68 (0.02, 1.33)** | **0.04** |  | 0.59 (-0.19, 1.36) | 0.14 |
| Paternal TICS-t, per 1 SD |  | 0.39 (-0.38, 1.16) | 0.32 |  | 0.14 (-0.65, 0.93) | 0.73 |  | **1.02 (0.42, 1.62)** | **<0.001** |
| Maternal education  (<12 yrs vs ≥12 yrs) |  | 0.24 (-1.26, 1.74) | 0.75 |  | 0.16 (-2.00, 2.32) | 0.88 |  | 0.89 (-1.18, 2.95) | 0.40 |
| Paternal education  (<12 yrs vs ≥12 yrs) |  | 0.49 (-1.28, 2.26) | 0.59 |  | 0.32 (-1.75, 2.39) | 0.76 |  | -0.67 (-2.84, 1.50) | 0.54 |
| Maternal nationality (German vs non-German) |  | -0.88 (-4.82, 3.06) | 0.66 |  | -0.25 (-4.38, 3.88) | 0.91 |  | -1.04 (-3.54, 1.45) | 0.41 |

SDQ-scores: lower scores indicate fewer social and emotional difficulties CI: confidence intervall; h: hours; vs: versus; w: week; yrs: years.

**Supplementary Table S9.** Adjustment variables in linear regression models estimating the association betwenn maternal employment after birth (n=5 trajectory classes) and health-related quality of life in children aged 4, 5, and 6 indexed with the total KINDL-score by gender. Ulm SPATZ Health Study, Germany.

|  |  | Difference in means (95% CI) in total KINDL-scores by child age | | | | | | | |
| --- | --- | --- | --- | --- | --- | --- | --- | --- | --- |
|  |  | 4 years | p |  | 5 years | p |  | 6 years | p |
| Exposure trajectory classes |  |  |  |  |  |  |  |  |  |
| BOYS |  |  |  |  |  |  |  |  |  |
| Had second child |  | **2.38 (0.39, 4.36)** | **0.02** |  | 1.72 (-0.86, 4.30) | 0.19 |  | 0.91 (-1.68, 3.50) | 0.49 |
| Got second child |  | **-3.78 (-6.92, -0.63)** | **0.02** |  | -1.54 (-5.46, 2.38) | 0.44 |  | -3.38 (-7.59, 0.83) | 0.12 |
| Regular childcare other than mother at 12 months (≥10h/w. vs <10h/w.) |  |  |  |  |  |  |  |  |  |
| Partner |  | 1.36 (-0.58, 3.29) | 0.17 |  | -0.26 (-2.86, 2.35) | 0.85 |  | **3.07 (0.62, 5.51)** | **0.01** |
| Center-based care |  | 1.06 (-1.99, 4.11) | 0.49 |  | 3.12 (-1.16, 7.39) | 0.15 |  | 1.70 (-1.98, 5.39) | 0.37 |
| Relative care |  | -1.23 (-3.81, 1.36) | 0.35 |  | -1.27 (-4.08, 1.55) | 0.38 |  | 1.73 (-1.29, 4.76) | 0.26 |
| Non-relative care |  | -3.31 (-8.60, 1.97) | 0.22 |  | -2.98 (-6.46, 0.50) | 0.09 |  | 2.14 (-2.58, 6.86) | 0.37 |
| Anxiety and depression symptoms pre/at birth |  |  |  |  |  |  |  |  |  |
| Maternal HADS, per 1 SD |  | -0.08 (-1.32, 1.15) | 0.90 |  | -0.45 (-1.76, 0.87) | 0.51 |  | -0.55 (-1.82, 0.72) | 0.39 |
| Maternal TICS-t, per 1 SD |  | **-3.31 (-4.48, -2.15)** | **<0.0001** |  | **-2.91 (-4.18, -1.63)** | **<0.0001** |  | **-2.14 (-3.24, -1.04)** | **0.0001** |
| Paternal TICS-t, per 1 SD |  | -0.62 (-1.60, 0.36) | 0.22 |  | -0.22 (-1.35, 0.92) | 0.71 |  | -0.83 (-1.97, 0.30) | 0.15 |
| Maternal education  (<12 yrs vs ≥12 yrs) |  | -1.27 (-3.56, 1.02) | 0.28 |  | -1.98 (-4.78, 0.82) | 0.17 |  | -1.30 (-3.93, 1.33) | 0.33 |
| Paternal education  (<12 yrs vs ≥12 yrs) |  | 0.52 (-1.57, 2.61) | 0.62 |  | -0.08 (-2.76, 2.59) | 0.95 |  | **3.10 (0.49, 5.71)** | **0.02** |
| Maternal nationality (German vs Non-German) |  | -0.06 (-4.07, 3.95) | 0.98 |  | 4.69 (-5.72, 15.09) | 0.38 |  | -2.22 (-5.95, 1.52) | 0.24 |
| GIRLS |  |  |  |  |  |  |  |  |  |
| Had second child |  | **2.96 (0.71, 5.20)** | **0.01** |  | **3.20 (1.01, 5.39)** | **0.004** |  | 1.94 (-0.82, 4.70) | 0.17 |
| Got second child |  | 1.94 (-0.86, 4.73) | 0.17 |  | 1.64 (-1.07, 4.34) | 0.24 |  | **3.80 (0.71, 6.89)** | **0.02** |
| Regular childcare other than mother at 12 months (≥10h/w. vs <10h/w.) |  |  |  |  |  |  |  |  |  |
| Partner |  | -1.12 (-3.23, 0.99) | 0.30 |  | -1.61 (-3.83, 0.61) | 0.16 |  | **-3.10 (-5.68, -0.52)** | **0.02** |
| Center-based care |  | -0.89 (-4.46, 2.68) | 0.63 |  | 0.58 (-2.99, 4.16) | 0.75 |  | 0.48 (-3.91, 4.87) | 0.83 |
| Relative care |  | 0.99 (-1.87, 3.85) | 0.50 |  | 0.69 (-2.09, 3.46) | 0.63 |  | **3.38 (0.46, 6.31)** | **0.02** |
| Non-relative care |  | -0.54 (-6.48, 5.39) | 0.86 |  | -4.76 (-9.73, 0.21) | 0.06 |  | -2.70 (-10.40, 4.99) | 0.49 |
| Anxiety and depression symptoms pre/at birth |  |  |  |  |  |  |  |  |  |
| Maternal HADS, per 1 SD |  | -0.07 (-1.37, 1.24) | 0.92 |  | -0.11 (-1.56, 1.35) | 0.88 |  | -0.41 (-1.92, 1.09) | 0.59 |
| Maternal TICS-t, per 1 SD |  | **-1.51 (-2.79, -0.23)** | **0.02** |  | **-2.41 (-3.79, -1.04)** | **<0.001** |  | -1.06 (-2.42, 0.30) | 0.13 |
| Paternal TICS-t, per 1 SD |  | -0.89 (-1.90, 0.13) | 0.09 |  | -0.73 (-1.78, 0.32) | 0.17 |  | -0.76 (-1.83, 0.32) | 0.17 |
| Maternal education  (<12 yrs vs ≥12 yrs) |  | 1.52 (-1.33, 4.38) | 0.30 |  | **3.09 (0.004, 6.18)** | **0.0497** |  | **4.26 (0.31, 8.21)** | **0.03** |
| Paternal education  (<12 yrs vs ≥12 yrs) |  | 0.05 (-2.70, 2.80) | 0.97 |  | -1.09 (-4.46, 2.27) | 0.52 |  | -2.42 (-6.29, 1.45) | 0.22 |
| Maternal nationality (German vs non-German) |  | -1.86 (-7.94, 4.22) | 0.55 |  | -5.29 (-10.80, 0.22) | 0.06 |  | -3.16 (-9.08, 2.76) | 0.30 |

KINDL-scores: higher scores indicate better health-related quality of life; CI: confidence intervall; h: hours; vs: versus; w: week; yrs: years.

**Supplementary Table S10.** Adjustment variables in linear regression models estimating the association betwenn maternal employment after birth (employment status 12 months after childbirth) and health-related quality of life in children aged 4, 5, and 6 indexed with the total KINDL-score by gender. Ulm SPATZ Health Study, Germany.

|  |  | Difference in means (95% CI) in total KINDL-scores by child age | | | | | | | |
| --- | --- | --- | --- | --- | --- | --- | --- | --- | --- |
|  |  | 4 years | p |  | 5 years | p |  | 6 years | p |
| Exposure employment status 12 months after childbirth |  |  |  |  |  |  |  |  |  |
| BOYS |  |  |  |  |  |  |  |  |  |
| Had second child |  | **2.26 (0.17, 4.35)** | **0.03** |  | 2.15 (-0.60, 4.89) | 0.13 |  | 0.28 (-2.42, 2.98) | 0.84 |
| Got second child |  | **-3.38 (-6.33, -0.43)** | **0.02** |  | -1.77 (-5.58, 2.04) | 0.36 |  | -2.20 (-6.18, 1.78) | 0.28 |
| Regular childcare other than mother at 12 months (≥10h/w. vs <10h/w.) |  |  |  |  |  |  |  |  |  |
| Partner |  | 1.29 (-0.67, 3.25) | 0.20 |  | 0.47 (-2.04, 2.99) | 0.71 |  | 2.05 (-0.41, 4.51) | 0.10 |
| Center-based care |  | 1.01 (-2.37, 4.39) | 0.56 |  | 4.31 (-1.05, 9.66) | 0.12 |  | 0.79 (-3.18, 4.75) | 0.70 |
| Relative care |  | -1.64 (-4.18, 0.90) | 0.21 |  | -1.18 (-4.41, 2.05) | 0.47 |  | 0.94 (-2.15, 4.02) | 0.55 |
| Non-relative care |  | -3.88 (-9.46, 1.71) | 0.17 |  | -3.44 (-8.11, 1.23) | 0.15 |  | 3.10 (-1.68, 7.89) | 0.20 |
| Anxiety and depression symptoms pre/at birth |  |  |  |  |  |  |  |  |  |
| Maternal HADS, per 1 SD |  | -0.07 (-1.33, 1.19) | 0.91 |  | -0.37 (-1.77, 1.04) | 0.61 |  | -0.60 (-1.90, 0.70) | 0.37 |
| Maternal TICS-t, per 1 SD |  | **-3.26 (-4.42, -2.11)** | **<0.0001** |  | **-2.76 (-4.09, -1.42)** | **<0.0001** |  | **-2.36 (-3.48, -1.25)** | **<0.0001** |
| Paternal TICS-t, per 1 SD |  | -0.55 (-1.56, 0.47) | 0.29 |  | -0.33 (-1.56, 0.90) | 0.59 |  | -0.61 (-1.82, 0.61) | 0.33 |
| Maternal education  (<12 yrs vs ≥12 yrs) |  | -1.33 (-3.63, 0.96) | 0.26 |  | -2.26 (-5.05, 0.52) | 0.11 |  | -0.95 (-3.39, 1.49) | 0.45 |
| Paternal education  (<12 yrs vs ≥12 yrs) |  | 0.77 (-1.39, 2.93) | 0.48 |  | 0.74 (-2.04, 3.51) | 0.60 |  | 2.16 (-0.43, 4.76) | 0.10 |
| Maternal nationality (German vs non-German) |  | 0.22 (-4.23, 4.67) | 0.92 |  | 6.02 (-6.29, 18.32) | 0.34 |  | **-4.35 (-8.25, -0.44)** | **0.03** |
| GIRLS |  |  |  |  |  |  |  |  |  |
| Had second child |  | **3.13 (0.89, 5.38)** | **0.006** |  | **2.89 (0.64, 5.14)** | **0.01** |  | 1.43 (-1.51, 4.37) | 0.34 |
| Got second child |  | 1.47 (-1.23, 4.16) | 0.28 |  | 1.50 (-1.12, 4.12) | 0.26 |  | **3.56 (0.49, 6.63)** | **0.03** |
| Regular childcare other than mother at 12 months (≥10h/w. vs <10h/w.) |  |  |  |  |  |  |  |  |  |
| Partner |  | -0.90 (-3.15, 1.34) | 0.43 |  | -1.16 (-3.29, 0.98) | 0.29 |  | -2.19 (-4.68, 0.30) | 0.08 |
| Center-based care |  | -0.52 (-4.60, 3.56) | 0.80 |  | 0.29 (-3.90, 4.48) | 0.89 |  | -0.01 (-5.63, 5.61) | 0.99 |
| Relative care |  | 0.88 (-2.36, 4.12) | 0.59 |  | 0.90 (-2.12, 3.93) | 0.56 |  | **3.31 (0.12, 6.51)** | **0.04** |
| Non-relative care |  | 0.82 (-4.59, 6.23) | 0.77 |  | -3.54 (-8.73, 1.65) | 0.18 |  | 1.01 (-2.86, 4.88) | 0.61 |
| Anxiety and depression symptoms pre/at birth |  |  |  |  |  |  |  |  |  |
| Maternal HADS, per 1 SD |  | 0.15 (-1.10, 1.40) | 0.82 |  | -0.03 (-1.48, 1.41) | 0.96 |  | 0.03 (-1.32, 1.37) | 0.97 |
| Maternal TICS-t, per 1 SD |  | **-1.51 (-2.78, -0.25)** | **0.02** |  | **-2.23 (-3.60, -0.85)** | **0.002** |  | -1.04 (-2.38, 0.31) | 0.13 |
| Paternal TICS-t, per 1 SD |  | -0.91 (-1.90, 0.09) | 0.08 |  | -0.96 (-2.02, 0.11) | 0.08 |  | -0.85 (-1.87, 0.17) | 0.10 |
| Maternal education  (<12 yrs vs ≥12 yrs) |  | 0.76 (-1.96, 3.49) | 0.58 |  | **3.06 (0.14, 5.99)** | **0.04** |  | **4.34 (0.20, 8.48)** | **0.04** |
| Paternal education  (<12 yrs vs ≥12 yrs) |  | 0.74 (-2.10, 3.58) | 0.61 |  | 0.05 (-2.82, 2.92) | 0.97 |  | -2.15 (-6.09, 1.78) | 0.28 |
| Maternal nationality (German vs non-German) |  | -1.96 (-9.27, 5.34) | 0.60 |  | -4.90 (-10.88, 1.08) | 0.11 |  | -3.47 (-10.22, 3.28) | 0.31 |

KINDL-scores: higher scores indicate better health-related quality of life; CI: confidence intervall; h: hours; vs: versus; w: week; yrs: years.
